# Inhibition of β-Amyloid Aggregation in Alzheimer’s Disease: The Key Role of (Pro)electrophilic Warheads

**DOI:** 10.1021/acsmedchemlett.2c00410

**Published:** 2022-10-10

**Authors:** Filippo Basagni, Marina Naldi, Tiziana Ginex, F. Javier Luque, Francesca Fagiani, Cristina Lanni, Matteo Iurlo, Massimo Marcaccio, Anna Minarini, Manuela Bartolini, Michela Rosini

**Affiliations:** † Department of Pharmacy and Biotechnology, Alma Mater Studiorum - University of Bologna, Via Belmeloro 6, 40126 Bologna, Italy; ‡ Department of Nutrition, Food Science, and Gastronomy, Institute of Biomedicine (IBUB) and Institute of Theoretical and Computational Chemistry (IQTCUB), University of Barcelona, Avinguda Prat de la Riba 171, 08921 Santa Coloma de Gramenet, Spain; § Department of Drug Sciences (Pharmacology Section), University of Pavia, V.le Taramelli 14, 27100 Pavia, Italy; ∥ Department of Chemistry “Giacomo Ciamician”, Alma Mater Studiorum - University of Bologna, Via Selmi 2, 40126 Bologna, Italy

**Keywords:** β-Amyloid, Alzheimer’s disease, Natural compounds, Covalent inhibition, Polyphenols

## Abstract

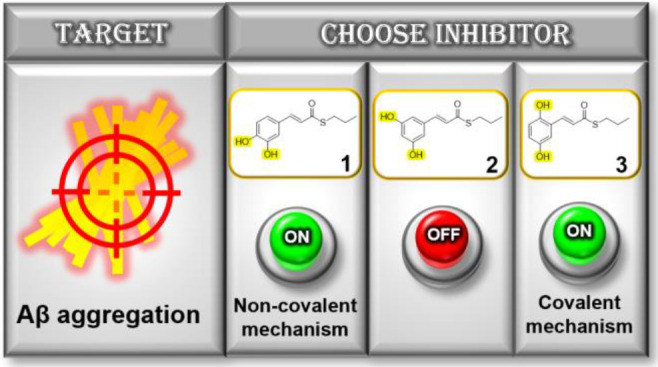

Catechols have been largely investigated as antiaggregating agents toward β-amyloid peptide. Herein, as a follow up of a previous series of hydroxycinnamic derivatives, we synthesized a small set of dihydroxy isomers for exploring the role of the reciprocal position of the two hydroxyl functions at a molecular level. *Para*- and *ortho*-derivatives effectively reduced amyloid fibrillization, while the *meta*-analogue was devoid of any activity in this respect. Electrochemical analyses showed that the antiaggregating potency correlates with the oxidation potential, hence indicating the proelectrophilic character as a prerequisite for activity. Interestingly, mass spectrometry studies and quantum mechanical calculations revealed different modes of action for active *para*- and *ortho*-derivatives, involving covalent or noncovalent interactions with β-amyloid. The distinctive mode of action is also translated into a different cytotoxicity profile. This work clearly shows how apparently minimal structural modifications can completely change the compound behavior and generate alternative mechanisms of action of proelectrophilic chemical probes.

Alzheimer’s disease (AD) is the most common protein misfolding disease, for which the amyloidogenic pathway represents a relevant feature. Amyloid oligomerization is a critical step of β-amyloid (Aβ) toxicity, as it shifts soluble nontoxic monomers into insoluble deposits through midterm stages of oligomerization and amyloid fibril formation. Soluble oligomers, rather than the insoluble end products of the amyloid cascade, contribute to Aβ synaptotoxicity.^1^ A variety of natural compounds have attracted the attention of the scientific community for their ability to interfere with protein misfolding.^2^ Several efforts have been made to understand their mode of action at a molecular level. Particularly, structure-dependent categorization of phenolic compounds has been performed suggesting both covalent and noncovalent mechanisms of amyloid binding.^3^

We previously reported a series of molecules based on the general formula **A** (Figure 1) as versatile chemical tools for investigating the molecular mechanisms potentially involved in Aβ damage.^4,5^

**Figure 1:**
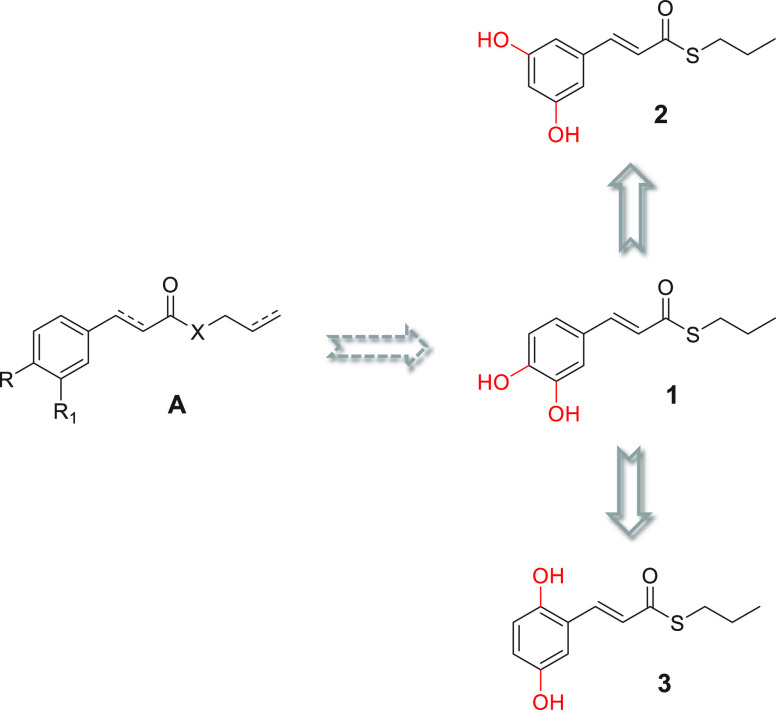
Drug design strategy.

Compound **1** emerged as the most potent antiaggregating agent (IC_50_ = 3.99 μM). SAR studies on those compounds revealed the importance of the thioester moiety and of the catechol function, which provided a peculiar “on–off” pattern of control of the antiaggregating effect.^4,5^

The presence of vicinal hydroxyl groups in the polyphenols baicalein, epigallocatechin, and (+)-taxifolin have been highlighted as a key structural feature for the inhibition of amyloidosis processes involving amylin, α-synuclein, and Aβ_42_.^3,6^ Thus, we sought to more deeply examine the role of the two vicinal hydroxyl functions of compound **1** and how they influence the mode of action of this class of compounds. To this aim, we synthesized a new set of derivatives where the hydroxyl function in position 4 of **1** was moved to position 5 or 6, achieving the corresponding *meta-* and *para-*derivatives **2** and **3** (Figure 1).

Synthesis of compounds **2** and **3** was carried out through a straightforward synthetic route with minor modifications to the procedure previously exploited to obtain compound **1**.^5^ As outlined in Scheme 1, selected dimethoxy benzaldehydes underwent a Knoevenagel condensation with malonic acid to give the corresponding α,β-unsaturated carboxylic acids **4** and **5**. Treatment of **4** and **5** with HOBt and EDC as activating agents and further coupling reaction with 1-propanethiol allowed obtaining intermediates **6** and **7**. Final treatment with BBr_3_ exerted demethylation, giving the final compounds **2** and **3**. ^1^H NMR spectra showed that compounds **1**–**3**, featuring a carbon–carbon double bond between the benzene ring and the carbonyl function, have an *E* configuration as indicated by the large spin coupling constants (around 16 Hz) of α-H and β-H on double bonds.

**Scheme 1:**
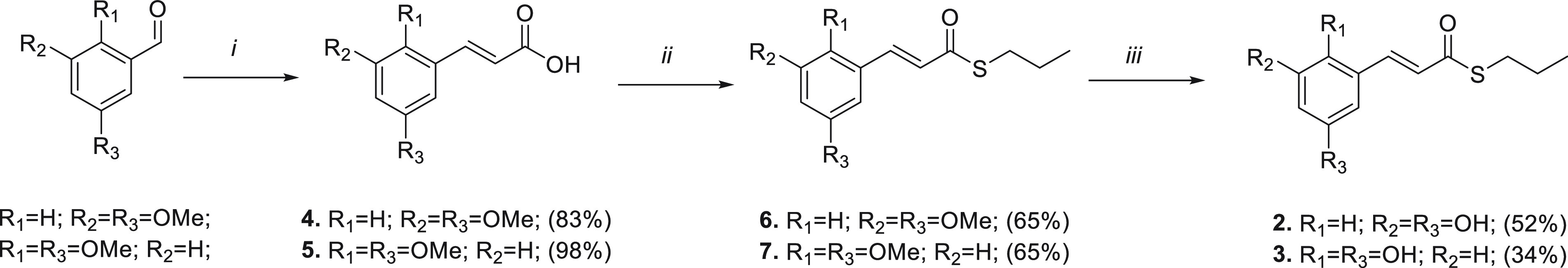
Synthesis of Thioester Isomers **2** and **3** Reagents and conditions: (*i*) malonic acid, pyridine, aniline, toluene, reflux, 4h; (*ii*) HOBt, EDC, 1-propanethiol, DCM, rt, o/n; (*iii*) BBr_3_ 1 M in DCM, DCM, 0 °C to rt, 2h.

The antiaggregating properties of the newly synthesized compounds were tested by a thioflavin-T (ThT) assay, a commonly used fluorimetric assay for *in vitro* screening of antiaggregating agents.^7^ Results from this evaluation confirmed that the reciprocal position of the two hydroxyl functions is a critical feature, since it strongly modulates the on–off switch of the antiaggregating efficacy. In particular, while *para*-hydroquinone **3**, as the corresponding *ortho*-quinone **1**, was able to effectively decrease amyloid fibrilization, the *meta*-derivative **2** was completely devoid of any inhibitory activity (Table 1). Compound **3** emerged as a strong antiaggregating agent, exerting an almost complete inhibition of amyloid aggregation when screened at equimolar concentration with Aβ_42_. **3** was only 2.5-fold less potent than the lead compound **1**. To further confirm these data and get further insight into the inhibition mechanism, the antiaggregating potencies of compounds **1**–**3** were also explored by mass spectrometry (MS) analysis. While ThT assay allows detecting/quantifying amyloid fibrils, MS allows detecting and quantifying the monomeric forms of Aβ_42_ and monitoring its disappearance as it is incorporated in growing Aβ oligomers and fibrils.^8^ Hence, in an MS assay a higher residual amount of monomeric Aβ_42_ is expected to be detected when an active inhibitor of the amyloid aggregation is co-incubated with Aβ_42_. Compounds **1** and **3** proved to exert such an action, confirming the ability to inhibit Aβ_42_ self-aggregation with the same trend outlined from the ThT assay, whereas compound **2** was inactive (Table 1).

**Table 1:** Antiaggregating Activities of Compounds **1**–**3** Compared with Corresponding Anodic Peaks (*E*_p_) and Standard Redox Potentials (*E*_1/2_)

	inhibition of Aβ_42_ self-aggregation			
cpd	% inhibition by ThT [I] = 50 μM^a^ (IC_50_ ± SEM/μM)	% inhibition by MS ± SD [I] = 50 μM^b^	*E*_p_(ox) (V)	*E*_1/2_(ox)^c^ (V)
**1**	>90 (3.99 ± 0.39)^d^	56.9 ± 3.5	0.19	0.10
**2**	<10	<10	0.76	0.66
**3**	>90 (10.2 ± 1.3)	43.6 ± 0.7	0.32	0.10

a Inhibition of Aβ_42_ 50 μM self-aggregation by [I] = 50 μM (tested compound/Aβ_42_ = 1/1). For compounds showing a % inhibition greater than 50%, the IC_50_ value was determined. Values are the mean of two independent experiments each performed in duplicate. SEM = standard error of the mean.

b Studies were performed by incubating Aβ_42_ samples under the assay conditions used for the ThT assay, with and without tested compounds at 50 μM (tested compound/Aβ_42_ = 1/1). Experiments were performed in duplicate. SD = standard deviation.

c Halfwave (*E*_1/2_) redox potential values estimated by digital simulation of the experimental voltammetric curves.

d IC_50_ already reported in ref (5).

The finding that *ortho*- and *para*-hydroquinones, but not the *meta*-hydroquinone, exert notable antiaggregating effects suggests that the oxidative properties of these compounds might be crucial for their inhibitory ability.

It is known that *ortho*- and *para*-hydroquinones can undergo full oxidation to semiquinone radicals and quinones while oxygen is reduced to hydrogen peroxide. Instead, resorcinol derivatives can convert to radical semiquinone but not to quinones.^9^ Cyclic voltammetry has been extensively exploited to depict the redox properties of catechols.^10^ Thus, to dissect the role of oxidative properties in a compound’s antiaggregating ability, the oxidation potential of isomers **1**–**3** was analyzed by performing cyclic voltammetry experiments, and the results were compared with functional biological activity. With potential scanned positively, catechols are converted into the corresponding *ortho*-benzoquinones, generating a characteristic anodic peak. On the reverse scan, the counterpart cathodic peak represents the reconversion of *ortho*-benzoquinones back to catechols. This is verified for the *ortho*- and *para*-derivatives, although affected by some degree of side reactivity following up the electron transfer (whose investigation is outside the scope of this work). An irreversible oxidation was observed for the *meta*-analogue **2** (Figure 2), in agreement with previous reports on resorcinol derivatives.^9^

**Figure 2:**
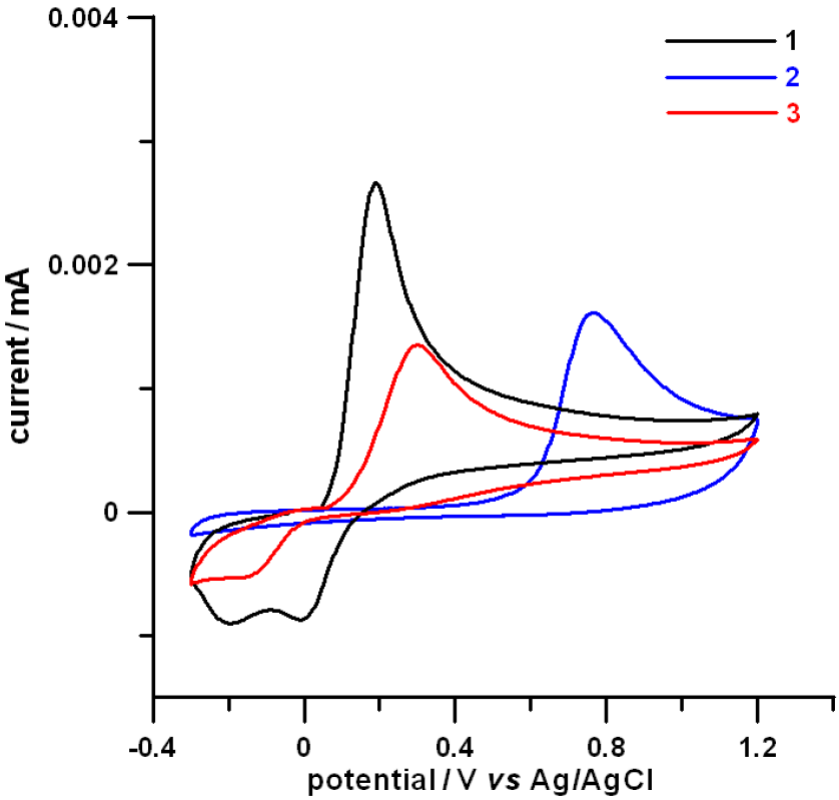
Cyclic voltammetric curves of **1** (black line), **2** (blue line), and **3** (red line) recorded in phosphate buffer 0.2 M electrolyte solution at a glassy carbon (GC) electrode and scan rate of 100 mV/s. The voltammetric curves represent the first potential sweep cycle as the subsequent cycles involve a passivation of the electrode surface by a film deposition, presumably formed by the oxidation byproducts.

Furthermore, a correlation was found by comparing the antiaggregating activities of the compounds with their anodic peak values (*E*_p_^an^), which match for smaller values to a higher inclination to oxidation, suggesting that a redox-controlled conversion to the active quinone form may plausibly be involved in the inhibition of amyloid aggregation. The same trend can be observed for the standard redox potentials (the experimental *E*_1/2_ values), instead of the peak potentials (Table 1).

The inhibitory activity of quinones toward Aβ assembly is well-documented,^11^ and both covalent and noncovalent mechanisms of inhibition have been proposed.^3,12^ Quinones are electrophilic species that can undergo nucleophilic addition from nucleophilic residues in proteins. However, the charge distribution of the quinone ring has been shown to exert a fundamental role in establishing a favorable dipole interaction between the central electron-poor quinone ring and the electron-rich peptide carbonyls as well as aromatic recognition sites within the amyloidogenic proteins.^13^

To achieve a conclusive answer on the possibility that quinone bioconversion is or is not followed by covalent interaction with β-amyloid peptide, the interaction of active compounds **1** and **3** with Aβ_42_ was studied by mass spectrometry. To this aim, Aβ_42_ samples were incubated (same assay conditions used for the ThT assay) with and without **1** or **3** at 50 μM (compound/Aβ_42_ = 1/1). No covalent adduct formation was detected when Aβ was co-incubated with **1**, strengthening the relevance of noncovalent interactions in triggering the biological response exerted by **1**. Conversely, the formation of a covalent adduct between Aβ_42_ and **3** was detected. In particular, the deconvoluted MS spectrum of Aβ_42_ at *t*_0_, that is immediately after the addition of compound **3** (Figure 3a), showed an intense signal at 4514 Da, which stands for the molecular weight of the monomeric native form of the peptide. Minor signals corresponding to the oxidized form of Aβ_42_ and its adduct with potassium ions were also detected.

**Figure 3:**
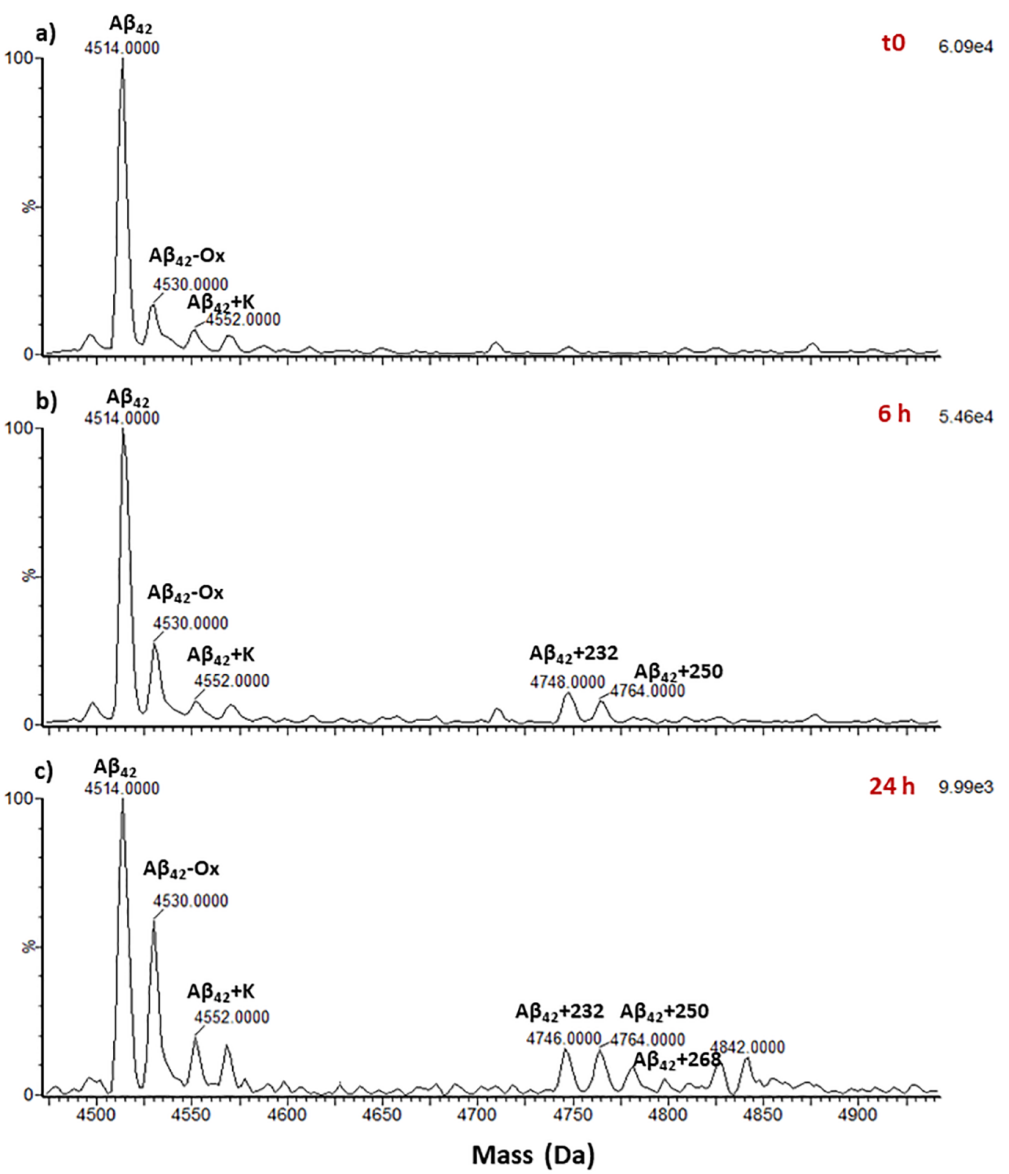
Deconvoluted mass spectra of Aβ_42_ after incubation for 0 h, corresponding to t0 (a), 6 h (b) and 24 h (c) with compound **3**. Aβ_42_+K stands for the adduct between Aβ_42_ and K^+^ while Aβ_42_-Ox refers to the oxidized form of the protein.

Co-incubation of Aβ_42_ with compound **3** led to the appearance of new species at higher molecular weights (Figure 3b,c). In particular, species characterized by a mass increment of 232 and 250 Da (signals at 4748 and 4764) were detected upon a 6 h incubation, while a further signal at 4782 (mass increment of 268 Da) was observed upon a 24 h incubation.

Further LC-MS/MS analyses revealed that Lys16 residue of Aβ_42_ is involved in the formation of both covalent adducts (Figure S1a,b). These results may lead to the hypothesis that the two adducts are related to each other and that A250 may result from a rearrangement of A232. In detail, the A232 adduct may form upon the double attack of the nucleophilic Lys16 on the oxidized quinone form of **3** (MW = 236 Da), affording a 4,7-dioxo-4,7-dihydro-1H-indole bicyclic structure. The formation of this adduct could first involve the attack of Lys16 to the *para*-quinone ring of **3**, followed by subsequent cyclization with the vinyl position (route a, Scheme 2) or, conversely, an initial addition to the vinyl position and a subsequent cyclization with the quinone moiety (route b, Scheme 2).

**Scheme 2:**
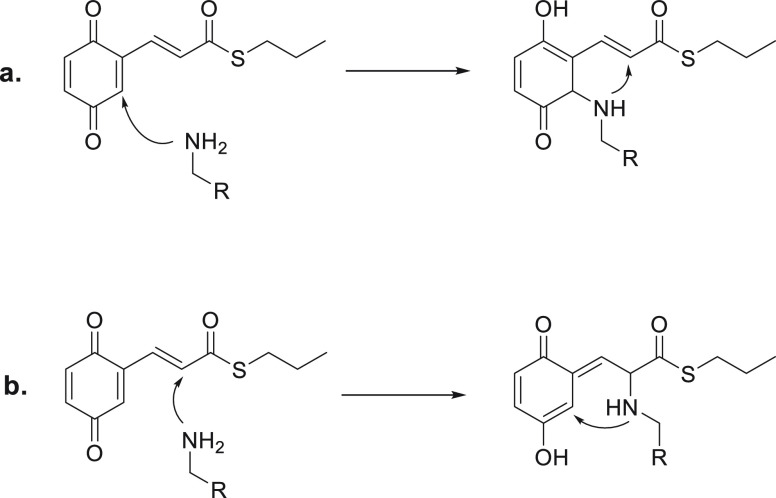
Hypothesized Double Attack of Lys16 to Oxidized **3** Possibly Justifying the Formation of A232 Adduct

To deepen this hypothesis and examine the molecular mechanism implicated in covalent adduct formation between compound **3** and Aβ_42_, quantum mechanical (QM) computations were performed. Given the lack of precise structural information on the inhibitor–Aβ complex, this study was performed using a reduced model, where the side chain of the Lys16 residue was simulated with methylamine. The free energy profile determined by density functional theory calculations (M062X/6-31G(d,p)) for the attack to the *para*-quinone ring of **3** reveals a barrier of 17.4 kcal/mol (relative to the separated reactants).

Additional calculations were also performed with the inclusion of a water molecule in order to check the potential effect in assisting the attack of methylamine to **3**, for which a decrease to 14.0 kcal/mol was observed (Figure 4). This can be ascribed to the formation of hydrogen bonds between the water molecule with the amine N (3.02 Å) and with the carbonyl oxygens of the *para*-quinone ring (3.22 Å) and the thioester moiety (3.17 Å). It is worth noting that the transition state (TS) is formed earlier than in the attack without the water molecule, as reflected in the distances from the N atom to the *para*-quinone carbon in the presence (2.02 Å) and absence (1.88 Å) of the water molecule.

**Figure 4:**
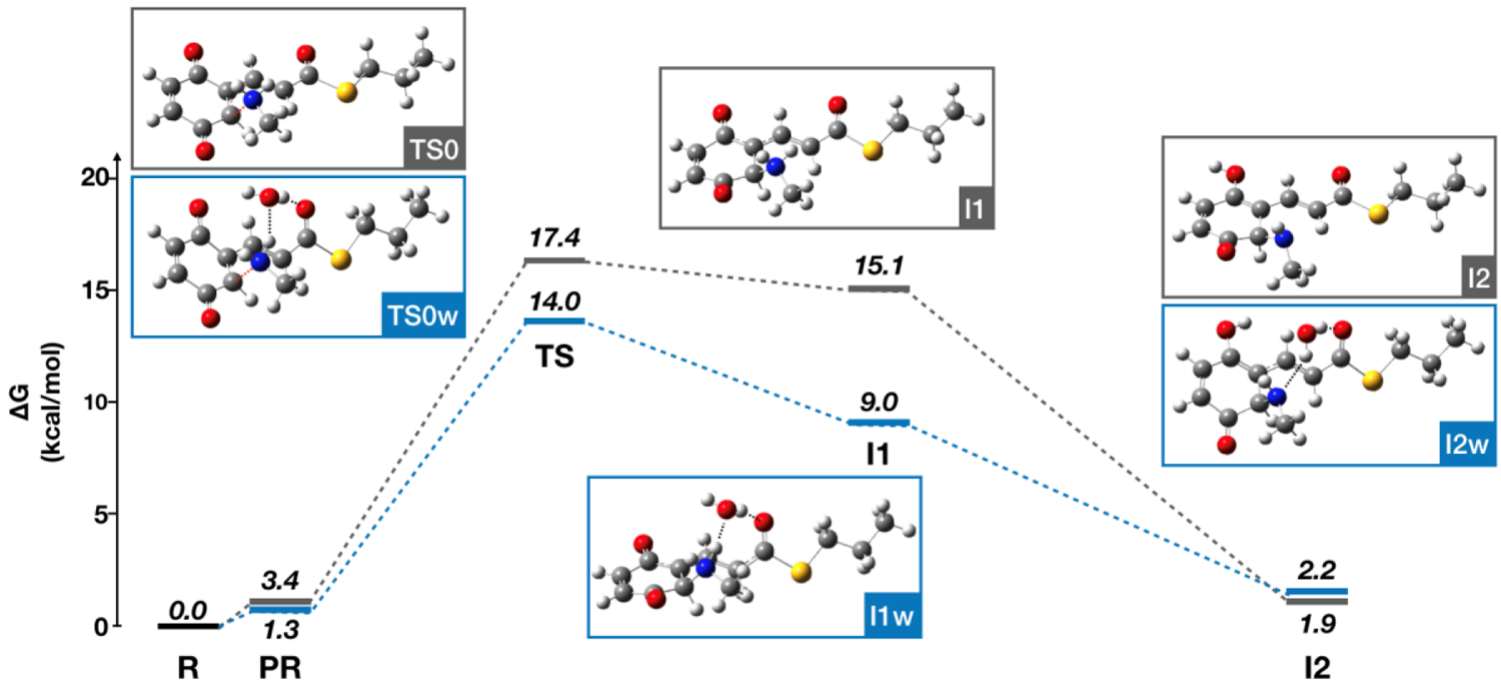
Free energy profiles in the gas phase determined from density functional theory calculations for the attack to **3** and performed using a reduced model where the side chain of Lys16 was simulated with methylamine and assisted (or not) by a water molecule.

The second hypothesis, i.e., an initial attack to the vinyl position (route b, Scheme 2), was found to be less favored, as the free energy barrier was estimated to be 25.3 and 19.0 kcal/mol in the absence and presence of a water molecule, respectively (Figure S2).

These results suggest that the adduct between Aβ_42_ and compound **3** could likely result from the attack of the nucleophilic Lys16 to the *para*-quinone ring of **3**. Keeping in mind the hydrophobic character of **3** (estimated logP close to 3.9 according to IEFPCM/MST continuum solvation calculations), this process may be promoted by the initial formation of a transient complex with Aβ monomers or dimers, as suggested for other catechol derivatives.^12,14^ The covalent adduct may subsequently evolve through an intramolecular cyclization, in conjunction with an oxidative process, to yield A232.

Finally, the interaction of Aβ_42_ and the *ortho*-quinone derivative **1** was also examined. In this case, the TS structures for the water-assisted nucleophilic attack of methylamine to the quinone and vinyl positions were destabilized by 20.5 and 24.4 kcal/mol (see Figure S3), which may contribute to explaining the lack of covalent modification upon incubation with this compound (see above).

It has been previously proven that Aβ can be easily modified by ROS at specific amino acidic residues, and the change of redox state can impair the biological behavior and the oligomerization process. Indeed, the oxidized form of Aβ_42_ (Aβ_42_ Ox) was shown to be less prone to aggregate than the native one (Aβ_42_ Native), accounting for its slower aggregation rate.^15,16^ In this respect, the peculiar profile of polyphenols, which can act as either antioxidant or pro-oxidant agents, offers an additional method of intervention in amyloid aggregation.^17^ A prototypical example is the pro-oxidant effect exerted by the natural polyphenol myricetin toward Aβ_42_.^16^

On this basis, we sought to verify whether the pro-electrophiles **1** and **3** could partially carry out their inhibitory activity through an oxidation-based mechanism. To estimate whether **1** or **3** may favor amyloid oxidation, variations in the content of Aβ_42_ Ox was monitored by MS analysis over time in the presence and absence of each inhibitor. As shown in Figure 5, Aβ_42_ oxidation increased over time when it was co-incubated with **3**, while no significant increase in Aβ_42_ Ox was detected when Aβ_42_ was co-incubated with **1**. This highlights a further peculiar different feature in the mode of action of these two compounds.

**Figure 5:**
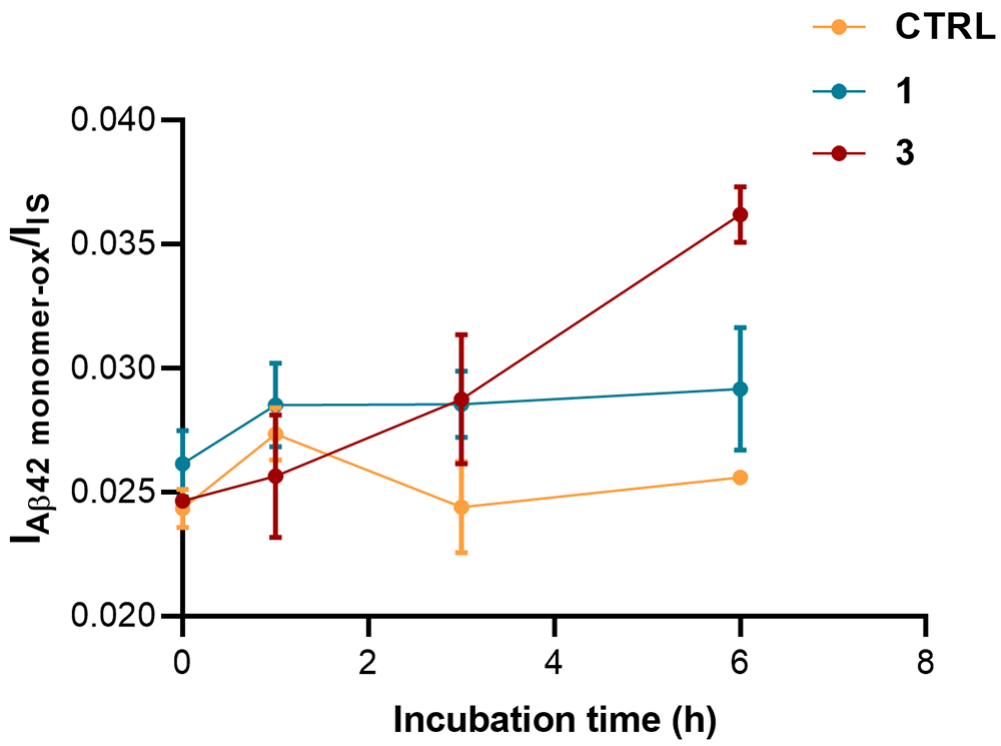
Evaluation of the oxidized form of Aβ_42_ in the absence and in the presence of **1** or **3** at equimolar concentration with Aβ_42_. The ratio between the signal corresponding to the oxidized form of Aβ_42_ and the signal of the internal standard (reserpine) is reported as a function of the incubation time.

Electrophilic features raise potential toxicity concerns, as covalent binding with nucleophiles in proteins is generally viewed as a basis for off-target interactions.^18^ Thus, cellular toxicity elicited by compounds **1**–**3** in SH-SY5Y human neuroblastoma cells was explored. Cells were treated with **1**–**3** at 5 and 10 μM. Cell viability was evaluated after a 24 h incubation by MTT assay, and results are reported in Figure 6. Electrophile **1** was well tolerated at tested concentrations, as was also the newly synthesized nonelectrophile **2**, which lacked any toxicity. Conversely, electrophile **3**, for which a covalent mode of action was shown, reduced cell viability in a dose-dependent manner, up to more than 50% when tested at 10 μM. Notably, a correlation could be found between cytotoxicity and the ability to covalently trap nucleophiles in proteins, whereas the (pro)electrophilic feature alone was shown to be less relevant in this respect.

**Figure 6:**
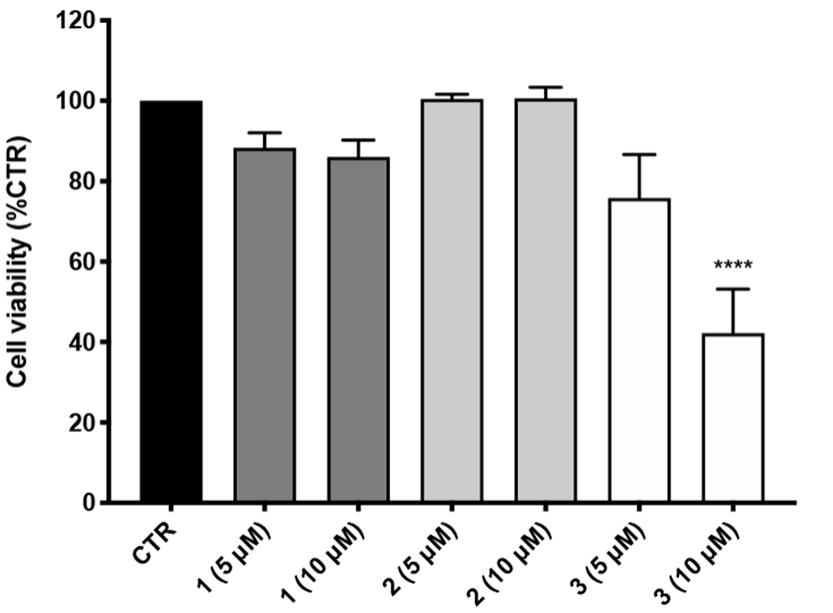
Cellular toxicity of compounds **1**–**3** in human neuroblastoma SH-SY5Y cells. Cells were treated with compounds **1**–**3** for 24 h at the concentrations of 5 and 10 μM. Cell viability was assessed by MTT assay. Data are expressed as percentage of cell viability ± SEM versus CTR; ****p < 0.0001 versus CTR; Dunnett’s multiple comparison test, *n* = 4.

In conclusion, we investigated the molecular mechanisms underpinning the antiaggregating activity of previously synthesized catechol-based compounds through the development of the corresponding *meta*- and *para*-dihydroxy-analogues of **1** and the study of their antiaggregating properties. Our results allowed disclosing the pro-electrophilic character as a prerequisite for compound activity. Indeed, the *ortho*- and *para*-derivatives, which can be easily oxidized to the corresponding quinones, strongly inhibited β-amyloid aggregation, whereas the *meta*-analogue was devoid of any relevant activity. Interestingly, MS analyses highlighted a different mode of action for compounds **1** and **3**, suggesting that the strong inhibitory activity of **3** arises from a variety of processes, including the formation of a covalent adduct with Lys16 residue of Aβ_42_ and an oxidation-based mechanism. Conversely, noncovalent interactions seem to represent the driving mechanism in the antiaggregating action of **1.** Notably, the different cytotoxic profile of compounds **1**–**3** is in line with the covalent or noncovalent mode of action, highlighting the concept that (pro)electrophilic features are not *per se* sufficient to covalently engage a target protein. Furthermore, the results highlight that apparently minimal structural modifications can generate alternative mechanisms of action and trigger distinctive toxicity profiles. These results support the view that electrophiles should not be *a priori* considered as structural alerts or toxicophores. A deep understanding and fine-tuning of chemical reactivity could be useful to explore the investigational and therapeutic potential of electrophile-modulator signals.

## Supporting Information Available

Materials and methods for synthetic procedures with characterization of final compounds, ThT- and MS-based studies, cyclic voltammetry analysis, and computational (with optimized geometries) and cellular experiments (PDF)

## Abbreviations

AD: Alzheimer’s disease
Aβ: amyloid-β peptide
BBr_3_: boron tribromide
DMF: *N*,*N*-dimethylformamide
DMSO: dimethyl sulfoxide
EDC: 1-ethyl-3-(3-(dimethylamino)propyl)carbodiimide hydrochloride
ESI-MS: electrospray ionization mass spectrometry
GST: glutathione S-transferase
HPLC: high-performance liquid chromatography
HOBt: 1-hydroxy benzotriazole hydrate
MW: molecular weight
NMR: nuclear magnetic resonance
ppm: parts per million
ROS: reactive oxygen species
SAR: structure–activity relationships
TLC: thin-layer chromatography
TMS: tetramethylsilane
ThT: thioflavin T; UV, ultraviolet.
