# Supplementary material for: Inhibition of β-Amyloid Aggregation in Alzheimer’s Disease: The Key Role of (Pro)electrophilic Warheads
Source: ACS Med Chem Lett. 2022 Oct 10;13(11):1812–8. doi: 10.1021/acsmedchemlett.2c00410 (PMC9661696; doi:10.1021/acsmedchemlett.2c00410)
Supplement: Supplementary file 1 — ml2c00410_si_001.pdf [file ml2c00410_si_001.pdf]

# Supporting Information

## Inhibition of β-amyloid aggregation in Alzheimer's disease: The key role of (pro)electrophilic warheads

Filippo Basagni [a], Marina Naldi [a], Tiziana Ginex [b], F. Javier Luque[b], Francesca Fagiani [c], Cristina Lanni [c], Matteo Iurlo [d], Massimo Marcaccio [d], Anna Minarini [a], Manuela Bartolini [a], Michela Rosini [a,*]

a) Department of Pharmacy and Biotechnology, Alma Mater Studiorum - University of Bologna, Via Belmeloro 6, 40126 Bologna, Italy

b) Department of Nutrition, Food Science, and Gastronomy, Institute of Biomedicine (IBUB) and Institute of Theoretical and Computational Chemistry (IQTCUB), University of Barcelona, Avinguda Prat de la Riba 171, 08921-Santa Coloma de Gramenet, Spain

c) Department of Drug Sciences (Pharmacology Section), University of Pavia, V.le Taramelli 14, 27100 Pavia, Italy

d) Department of Chemistry "Giacomo Ciamician", Alma Mater Studiorum - University of Bologna, Via Selmi 2, 40126 Bologna, Italy

**Table of contents:**

Experimental section page S2

Figures S1-S3 page S5

NMR spectra for final compounds **2** and **3** page S7

Optimized geometries page S9

Bibliography page S24

Corresponding author: Michela Rosini - Department of Pharmacy and Biotechnology, Alma Mater Studiorum - University of Bologna, Via Belmeloro 6, 40126 Bologna, Italy; Email: michela.rosini@unibo.it, Phone: +39 051 2099722

**Experimental section**

No unexpected or unusually high safety hazards were encountered in all experiments of this study.

**Chemistry.** Chemical reagents were purchased from Merck, TCI and Fluorochem. Nuclear magnetic resonance spectra (NMR) were recorded at 400 MHz for $^1$H and 100 MHz for $^{13}$C on Varian VXR 400 spectrometer in $CDCl_3$, DMSO-*d*$_6$ or $CD_3OD$ as solvents. Chemical shifts ($\delta$) are given in ppm from tetramethylsilane (TMS) with the solvent resonance as internal standard ($CDCl_3$: $\delta$ 7.26, DMSO-*d*$_6$: $\delta$ 2.50, $CD_3OD$: $\delta$ 3.31 for $^1$H NMR and $CDCl_3$: $\delta$ 77.16, DMSO-*d*$_6$: $\delta$ 39.52, $CD_3OD$: $\delta$ 49.00 for $^{13}$C NMR). For $^1$H NMR, data are reported as follows: chemical shift, multiplicity (s = singlet, d = doublet, dd = double of doublets, t = triplet, q = quartet, m = multiplet, p = pentet, dt = doublet of triplets, td = triplet of doublets, tt = triplet of triplets, qd = quartet of doublets, br s = broad singlet), coupling constants (Hz) and integration. Chromatographic separations were performed on silica gel columns by flash or gravity column (Kieselgel 40, 0.040-0.063 mm; Merck) chromatography. Reactions were followed by thin-layer chromatography (TLC) on Merck (0.25 mm) glass-packed pre-coated silica gel plates (60 F254) that were visualized in an iodine chamber, or with a UV lamp, $KMnO_4$, or bromocresol green. All the names were attributed by Chem BioDraw Ultra 20.0. Final compounds mass spectra were recorded on a Xevo G2-XS QTof apparatus with electrospray ionization (ESI) in positive mode. All final compounds were pure >95% as determined by HPLC. HPLC analyses were performed under reversed-phase conditions on a Phenomenex Jupiter C18 (150x4.6 mm I.D.) column, using as the mobile phase a binary mixture of $H_2O$/acetonitrile (50/50, v/v) with UV detection at λ = 302 nm and a flow rate of 1 mL/min. Analyses were performed on a liquid chromatograph model PU-1585 UV equipped with a 20 µL loop valve (Jasco Europe, Italy). Compounds **1** was prepared as reported in reference [1].

*General procedure for the synthesis of compounds **4** and **5**.* Malonic acid (1.1 eq) was added to a solution of aniline (0.03-0.05 mL), pyridine (1.5 eq) and selected benzaldehyde (1 eq) in 2-4 mL of toluene and the mixture stirred at reflux for 4h. Once cooled to room temperature, HCl 3N was added until pH=1. The obtained precipitate was filtered, washed with HCl 3N and dried.

*(E)-3-(3,5-dimethoxyphenyl)acrylic acid* (**4**). Compound **4** was synthesized from 3,5-dimethoxybenzaldehyde (500 mg, 3.01 mmol) and obtained as white powder (520 mg, 83%). $^1$H NMR (400 MHz, $CDCl_3$) δ 7.69 (d, *J* = 16 Hz, 1H), 6.68-6.67 (m, 2H), 6.51-6.50 (m, 1H), 6.41 (d, *J* = 16 Hz, 1H), 3.81 (s, 6H). $^{13}$C NMR (100 MHz, $CDCl_3$) δ 171.72, 161.03 (2C), 147.06, 135.85, 117.66, 106.19 (2C), 103.01, 55.44 (2C).

*(E)-3-(2,5-dimethoxyphenyl)acrylic acid* (**5**). Compound **5** was synthesized from 2,5-dimethoxybenzaldehyde (400 mg, 2.41 mmol) and obtained as yellowish powder (490 g, 98%). $^1$H NMR (400 MHz, DMSO-d$_6$) δ 12.27 (br s, 1H), 7.77 (d, *J* = 16 Hz, 1H), 7.22-7.21 (m, 1H), 7.00-6.94 (m , 2H), 6.52 (d, *J* =16 Hz, 1H), 3.77 (s, 3H), 3.71 (s, 3H). $^{13}$C NMR (100 MHz, DMSO-d$_6$) δ 167.78, 153.12, 152.01, 138.37, 122.94, 119.50, 117.46, 112.94, 112.50, 56.01, 55.48.

*General procedure for the synthesis of compounds **6** and **7**.* To a solution of corresponding acid **4** and **5** (1 eq) in 1.5-3.5 mL of dichloromethane at 0°C under nitrogen atmosphere, HOBt (1.3 eq) and EDC (1.3 eq) were added. Reaction mixture was left stirring at this temperature for 30 min. After confirming the complete conversion of starting material in activated complex, selected thiol (4 eq) was added and the reaction mixture left stirring overnight at room temperature. After evaporation of the solvent, the crude was purified through flash column chromatography using petroleum ether: ethyl acetate as mobile phase.

*S-propyl (E)-3-(3,5-dimethoxyphenyl)prop-2-enethioate* (**6**). Compound **6** was synthesized from **4** (520 mg, 2.50 mmol) and 1-propanethiol (0.91 mL, 9.99 mmol). Compound was eluted with petroleum ether: ethyl acetate (9:1), which afforded **6** as colourless oil (430 mg, 65%). $^1$H NMR (400 MHz, $CDCl_3$) δ 7.50 (d, *J* = 16 Hz, 1H), 6.67-6.64 (m, 3H), 6.49-6.47 (m, 1H), 3.80 (s, 6H), 2.98 (t, *J* = 7.2 Hz, 2H), 1.68-1.61 (m, 2H), 0.99 (t, *J* = 7.4 Hz, 3H). $^{13}$C NMR (100 MHz, $CDCl_3$) δ 189.90, 161.00, 140.17, 136.03, 125.58, 106.15, 102.79, 55.44, 30.86, 22.99, 13.38.

*S-propyl (E)-3-(2,5-dimethoxyphenyl)prop-2-enethioate* (**7**). Compound **7** was synthesized from **5** (300 mg, 1.44 mmol) and 1-propanethiol (0.52 mL, 5.76 mmol). Compound was eluted with petroleum ether: ethyl acetate (9.2:0.8), which afforded **7** as yellowish oil (250 mg, 65%). $^1$H NMR (400 MHz, $CDCl_3$) δ 7.89 (d, *J* = 15.8 Hz, 1H), 7.04-7.03 (m, 1H), 6.92-6.89 (m, 1H), 6.85-6.82 (m, 1H), 6.77 (d, *J* = 15.8 Hz, 1H), 3.83 (s, 3H), 3.78 (s, 3H), 2.98 (t, *J* = 7.2 Hz, 2H), 1.69-1.64 (m, 2H), 1.01 (t, *J* = 7.4 Hz, 3H). $^{13}$C NMR (100 MHz, $CDCl_3$) δ 190.42, 153.58, 153,32, 135.66, 126.01, 123.75, 117.50, 113.40, 112.54, 56.16, 55.88, 30.88, 23.14, 13.49.

*General procedure for the synthesis of compounds **2** and **3**.* A solution of dimethoxy analogues **6** and **7** (1 eq) in 0.2-0.6 mL of anhydrous dichloromethane was cooled at 0°C. Boron tribromide 1M in dichloromethane (2.3 eq) was added to the solution and the reaction was left stirring at the same temperature until completion. 6-10 mL of cold water were added to terminate the reaction and the mixture extracted with ethyl acetate (3 x 8 mL). Organic phases, once

reunited, were dried with anhydrous sodium sulphate, concentrated in vacuo and the crude obtained purified by column chromatography using dichloromethane: methanol as mobile phase.

*S-propyl (E)-3-(3,5-dihydroxyphenyl)prop-2-enethioate* (**2**). Compound **2** was synthesized from **6** (430 mg, 1.61 mmol). Compound was eluted with dichloromethane: methanol (9.5:0.5), which afforded **2** as yellow powder (200 mg, 52%). $^1$H NMR (400 MHz, $CD_3OD$) δ 7.41 (d, *J* = 15.8 Hz, 1H), 6.67 (d, *J* = 15.8 Hz, 1H), 6.51 (s, 2H), 6.33-6.32 (m, 1H), 2.95 (t, *J* = 7.2 Hz, 2H), 1.66-1.59 (m, 2H), 0.99 (t, *J* = 7.2 Hz, 3H). $^{13}$C NMR (100 MHz, $CD_3OD$) δ 191.65, 160.12 (2C), 141.97, 137.21, 125.76, 107.76 (2C), 106.16, 31.56, 24.18, 13.58. HRMS (ESI+) calcd for $C_{12}H_{14}O_3S$ $[M+H]^+$ 239.0736, found 239.0744.

*S-propyl (E)-3-(2,5-dihydroxyphenyl)prop-2-enethioate* (**3**). Compound **3** was synthesized from **7** (220 mg, 0.83 mmol). Compound was eluted with dichloromethane: methanol (9.6:0.4), which afforded **3** as yellow powder (66 mg, 34%). $^1$H NMR (400 MHz, $CD_3OD$) δ 7.82 (d, *J* = 16 Hz, 1H), 6.89-6.88 (m, 1H), 6.79 (d, *J* = 16 Hz, 1H), 6.69-6.68 (m, 2H), 2.94 (t, *J* = 7.4 Hz, 2H), 1.65-1.60 (m, 2H), 0.99 (t, *J* = 7.4 Hz, 3H). $^{13}$C NMR (100 MHz, $CD_3OD$) δ 190.87, 150.60, 149.93, 136.23, 124.07, 121.14, 119.10, 116.54, 113.38, 30.07, 22.84, 12.17. HRMS (ESI+) calcd for $C_{12}H_{14}O_3S$ $[M+H]^+$ 239.0736, found 239.0742.

**Cyclic voltammetry study.** Electrochemical experiments were carried out in an airtight single-compartment glass cell described elsewhere[2] by using glassy carbon (GC) as working electrode, a platinum spiral as counter electrode and a silver spiral surface modified by silver chloride as a reference electrode. For the measurements in aqueous solutions a 0.2M phosphate buffer was used as electrolyte solution. All the $E_{1/2}$ potentials have been directly obtained from CV curves as averages of the cathodic and anodic peak potentials for one-electron reversible peaks and by digital simulation for those processes either closely spaced in multielectron voltammetric peaks or for the irreversible electron transfers. The $E_{1/2}$ values are referred to an Ag/AgCl (KCl, 3M). The GC working electrode was a 3 mm diameter disk that was mechanically polished by an alumina slurry in water. Voltammograms were recorded with a Biologic potentiostat model SP-300. Digital simulations of the cyclic voltammetric curves were carried out either by Antigona or DigiSim 3.0.

**Sample preparation for $A\beta_{42}$ self-aggregation.** 1,1,1,3,3,3-Hexafluoro-2-propanol (HFIP)-pretreated $A\beta_{42}$ samples (Bachem AG, Switzerland) were resolubilized with a $CH_3CN$/0.3 mM $Na_2CO_3$/250 mM NaOH (48.4:48.4:3.2) mixture to have a stable stock solution ([$A\beta_{42}$] = 500 µM).[3] Tested inhibitors were dissolved in MeOH and diluted in the assay buffer. Experiments were performed by incubating the peptide diluted in 10 mM phosphate buffer (pH 8.0) containing 10 mM NaCl at 30°C (Thermomixer Comfort, Eppendorf, Italy) for 24 h (final Aβ concentration = 50 µM) with and without inhibitor.

**Inhibition of $A\beta_{42}$ self-aggregation by ThT.** Inhibition studies were performed by incubating $A\beta_{42}$ samples in the assay conditions reported above, with and without tested inhibitors. Inhibitors were first screened at 50 µM in a 1:1 ratio with $A\beta_{42}$. To quantify amyloid fibril formation, the ThT fluorescence method was used.[4] After incubation, samples were diluted to a final volume of 2.0 mL with 50 mM glycine-NaOH buffer (pH = 8.5) containing 1.5 µM ThT. A 300 s-time scan of fluorescence intensity was carried out ($\lambda_{exc}$ = 446 nm; $\lambda_{em}$ = 490 nm), and values at plateau were averaged after subtracting the background fluorescence of 1.5 µM ThT solution. Blanks containing inhibitor and ThT were also prepared and evaluated to account for quenching and fluorescence properties. The fluorescence intensities were compared and the % inhibition was calculated. For selected compounds the $IC_{50}$ value was also determined. To this aim four increasing concentrations were tested. $IC_{50}$ value was obtained from the % inhibition vs log[inhibitor] plot.

**Analysis of intact protein (Top-down analysis).** ***Sample preparation.*** Studies were performed by incubating $A\beta_{42}$ samples under the assay conditions used for the ThT assay, with and without tested compounds (**1**-**3**) at 50 µM (tested compound/$A\beta_{42}$ = 1/1). Samples were incubated at 30°C for 24 h. At selected time points (0, 1, 3, 6 and 24h) 5 µL-aliquots of mixture were collected, diluted to 25 µL with reserpine 20 µg/mL (IS) in $H_2O$/MeOH (50/50; v/v) and subjected to LC-MS analysis. Experiments were performed in duplicate.

***LC-MS analysis.*** LC-MS analyses were performed by an Agilent 1200 Series (Walbronn, Germany) coupled with an ESI-Q-ToF mass spectrometer equipped with a Z-spray ion source (Micromass, Manchester, UK). Flow injection analyses were performed by employing $H_2O$/AcCN/FA (70/30/0.1; v/v/v) as mobile phase at the flow rate of 0.1 mL/min. The injection volume was 5 µL. The capillary voltage was set at 3000 V, the cone voltage was 35 V, the capillary temperature was 120°C, while the desolvation temperature was 300°C. Mass spectra were recorded in total ion current (TIC), within 500 and 2000 *m/z*, in positive polarity. $A\beta_{42}$ baseline subtracted spectrum (m/z 650−1700) was deconvoluted onto a true mass scale using the maximum entropy (MaxEnt1)-based software supplied with MassLynx

4.1 software. The output parameters were mass range: 4000–5000 Da and resolution: 2 Da/channel. The uniform Gaussian model was used, with 0.65 Da width at half height.

***Assessment of the inhibitory capacity.*** The ratio between intensity of the $[M+5H]^{5+}$ charge state of $A\beta_{42}$ monomer (at m/z 903.8) and the intensity of the internal standard (m/z at 609.4), namely $I_{A\beta42}/I_{IS}$, was used to assess the residual amount of $A\beta_{42}$ monomer. The $I_{A\beta42}/I_{IS}$ ratio at t0 was considered as 100% of the monomer content. $I_{A\beta42}/I_{IS}$ values at 24h were also calculated to assess the residual amount of monomer (R) as difference from the $I_{A\beta42}/I_{IS}$ value at 24h and t0. Percent inhibition calculated as follows:

% inhibition = $100-(100xR_{inh}/R_0)$

where $R_{inh}$ and $R_0$ are the residual amount of amyloid monomer in the presence and in the absence of inhibitor.

**Bottom-up analysis of $A\beta_{42}$ upon incubation with compound 3.** ***Sample preparation.*** $A\beta_{42}$ 50 µM alone and co-incubated with **3** 50 µM were incubated for 6 h and digested overnight at 37°C upon dilution 1:2 with a solution of trypsin in ammonium bicarbonate 20 mM, pH 8; the final $A\beta_{42}$/trypsin ratio was 100/1 (w/w).

***LC-MSMS analysis.*** $A\beta_{42}$ tryptic digest (10 µL) was analyzed by an Agilent 1100 Series HPLC system (Walbronn, Germany) coupled with a Q-ToF mass spectrometer (Micromass, Manchester, UK) equipped with a Z-spray ion source. Analyses were performed on a C18 column (Aeris peptide XB-C18; 150 × 2.1 mm; 3.5 µm; Phenomenex). Mobile phases A [water/acetonitrile/FA (99/1/0.1, v/v/v)] and B [water/acetonitrile/FA (1/99/0.1, v/v/v)] were used to develop a gradient. The solvent gradient was set as follows: A–B from (98:8, v/v) to (40:60, v/v) in 20 min; (40:60, v/v) for 2 min. The column was equilibrated with the mobile phase composition of the starting conditions for 10 min before the next injection. The ESI-Q-ToF source temperature was set at 100°C, the capillary voltage at 3.0 kV, and the cone voltage at 35 V. Peptide ions within a m/z 400–1700 survey scan mass range were analyzed for subsequent fragmentation. 2+, 3+, and 4+ charged ions exceeding a threshold abundance (TIC value: 10 counts/s) were selected for MS/MS analyses. From a single survey scan, 4 ions were selected for subsequent fragmentation. Scan returns to mass survey mode when the ion intensity falls below 5 counts/s or after 8 s. Scan time was 1 s for the parent ion and 1 s for the MS/MS ions. The collision energy was selected using charge state recognition.

**Computational Methods**. QM computations were performed to discern the feasibility of the nucleophilic addition of the methylamine to the *para*-quinone ring as well as to the vinyl position. Moreover, calculations were also performed to characterize the energetics of the addition to the *ortho*-quinone derivative.

All computations were performed at the M06-2X/6-31G(d) level.[5] Full geometry optimizations were performed for the different model systems, and in all cases the vibrational frequencies were analyzed to confirm the nature of the stationary points (no imaginary frequency for minimum energy structures, and 1 imaginary frequency for the transition states; atomic coordinates for the optimized structures of the stationary points are provided in the Supporting Information). The free energy profiles were obtained by adding thermal and entropy corrections in the gas phase. Hydration effects were determined by using the IEFPCM/MST method at the B3LYP/6-31G(d) level.[6]

All calculations were performed with a locally modified version of Gaussian 16.[7]

**Sample preparation for cellular experiments.** Compounds **1**-**3** were solubilized in dimethyl sulfoxide (DMSO) (at stock concentrations) and frozen (–20°C) in aliquots that were diluted immediately prior to use. For each experimental setting, one stock aliquot was thawed out and diluted to minimize compound damage due to repeated freeze and thaw cycles. The final concentration of DMSO in culture medium was < 0.1%.

**SH-SY5Y Cell Cultures.** Human neuroblastoma SH-SY5Y cells, purchased from the European Collection of Cell Cultures (ECACC No. 94030304), were cultured in a medium with equal amounts of Eagle's minimum essential medium and Nutrient Mixture Ham's F-12, supplemented with 10% heat-inactivated fetal bovine serum (FBS), 2 mM glutamine, 0.1 mg/mL streptomycin, 100 IU·mL penicillin and non-essential amino acids at 37°C in 5% $CO_2$-containing, and 95% air atmosphere. All culture media, supplements and FBS were purchased from Sigma-Aldrich (Merck KGaA, Darmstadt, Germany). The experiments were carried out on passages 7–15. Cells were treated as reported in the figure legend.

**Cell Viability.** The mitochondrial dehydrogenase activity that reduces 3-(4,5-dimethylthiazol-2-yl)-2,5-diphenyl-tetrazolium bromide (MTT) (Sigma Aldrich, Merck KGaA, Darmstadt, Germany) was used to determine cell viability, using a quantitative colorimetric assay.[8] At day 0, SH-SY5Y cells were plated in 96-well plates at a density of 20 x $10^3$ viable cells per well. After treatment, according to the experimental setting, cells were exposed to an MTT solution (1 mg/mL) in complete medium. After 4 hours of incubation with MTT, cells were lysed with DMSO and cell viability was

quantified by reading absorbance at 570 nm wavelength, using Synergy HT multi-detection microplate reader (Bio-Tek, Winooski, VT, USA).

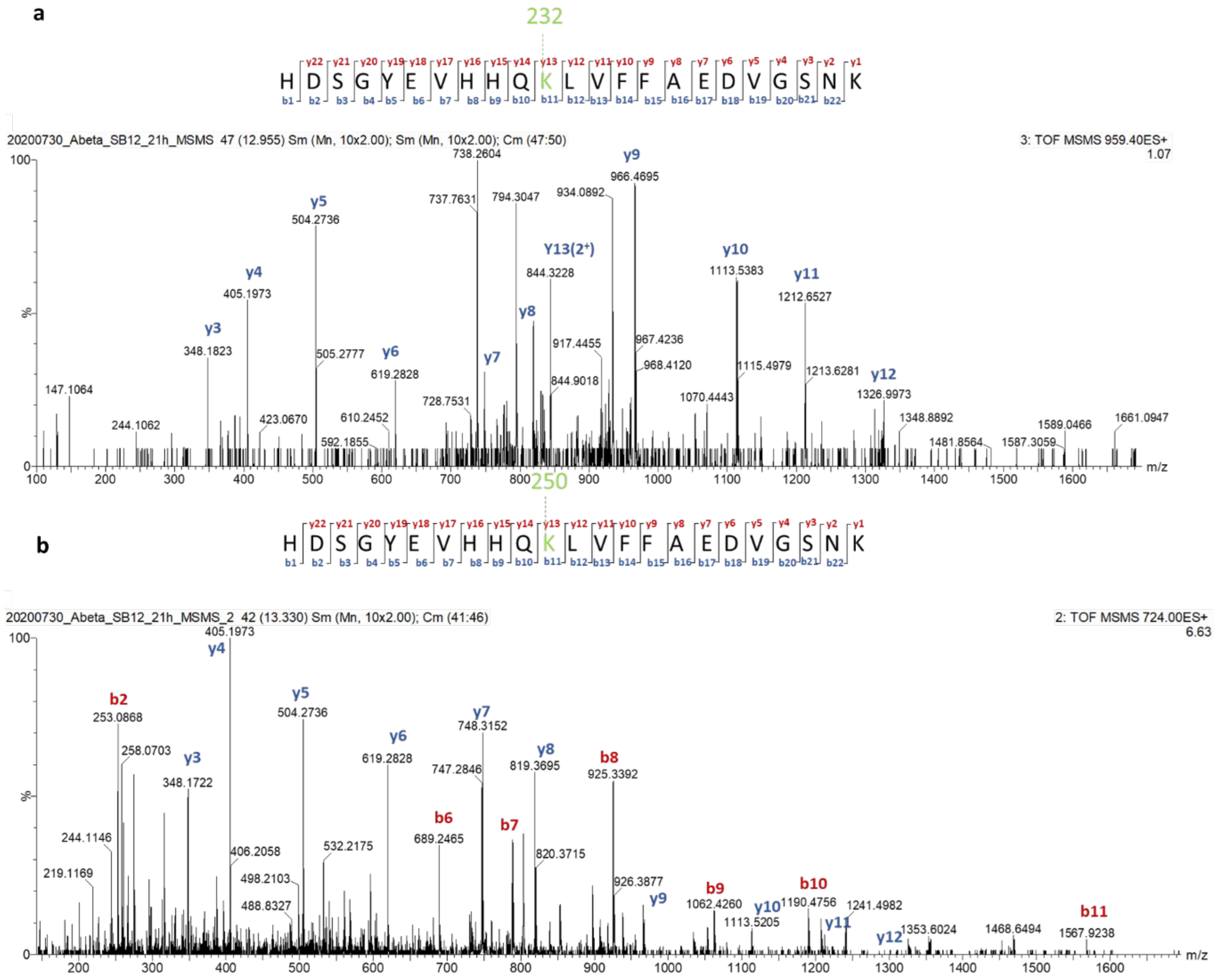


**Figure S1.** Bottom-up analysis of A$\beta_{42}$. MS/MS spectra of peptide 6-28 carrying a modification characterized by a mass increment of 232 (a) and 250 Da (b) at the level of A$\beta_{42}$ Lys16. In the mass spectra the identified fragments are labelled with the letter y or b, depending on the type of fragment, and with a number indicating the position of the fragmentation on the peptide sequence.

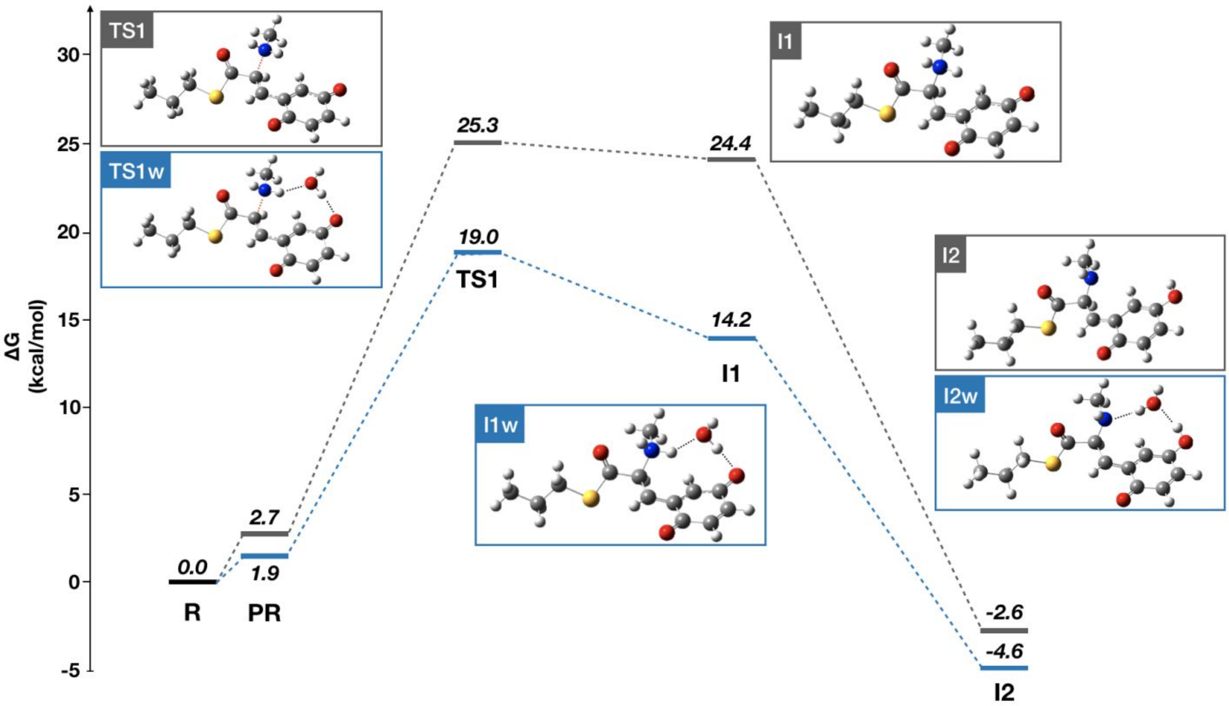


**Figure S2.** Free energy profiles in gas phase determined from density functional theory calculations for the attack to the vinyl position of **3** (see also route b, scheme 2 in the main text) and performed using a reduced model where the side chain of Lys16 was simulated with methylamine and assisted (or not) by a water molecule.

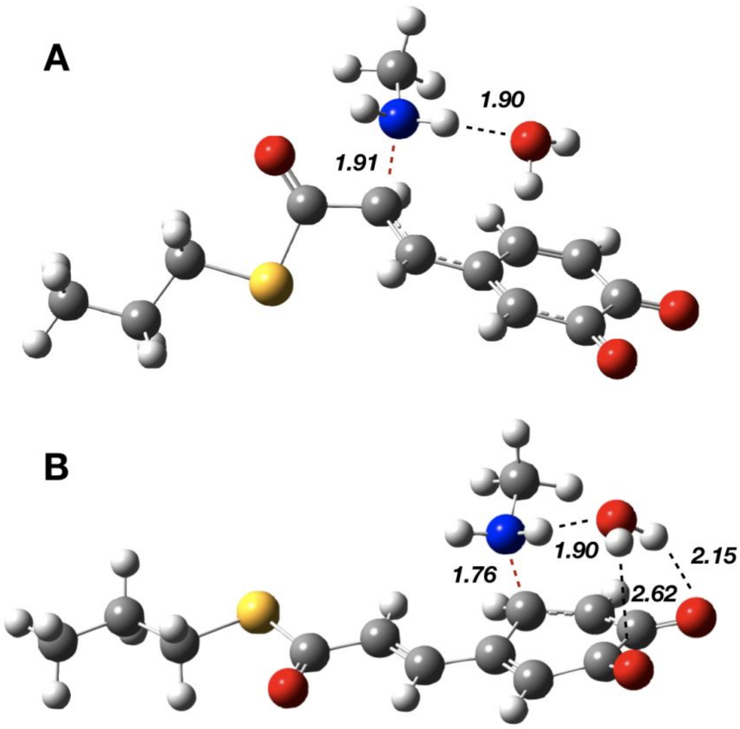


**Figure S3**. Transition (TS) structures from quantum mechanics (QM) calculations relative to the *water-assisted* addition of the methylamine to the vinyl (**A**) and to the quinone (**B**) moiety of the ortho-quinone derivative **1**.

$^{1}H$ NMR and $^{13}C$ NMR spectra of compounds **2** and **3**

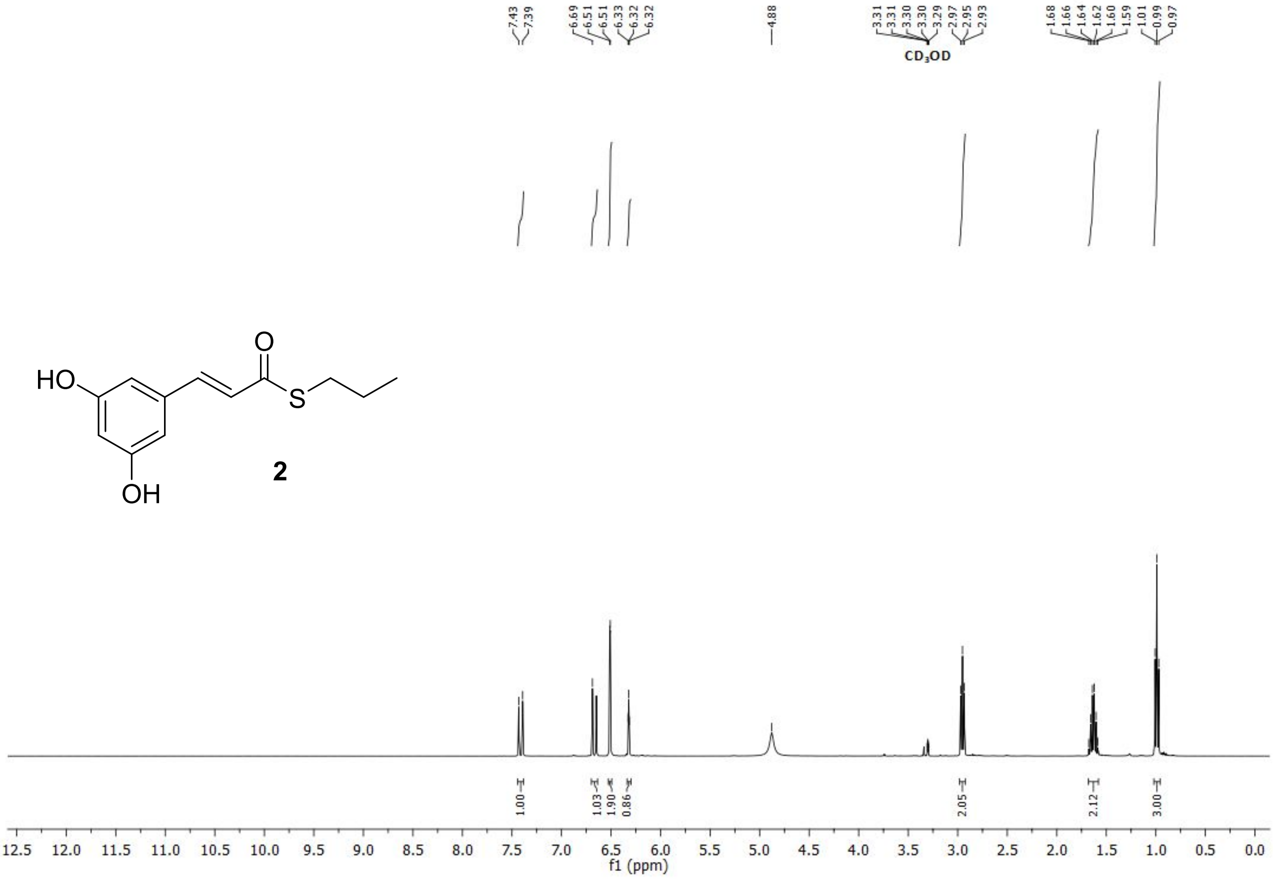


**2**

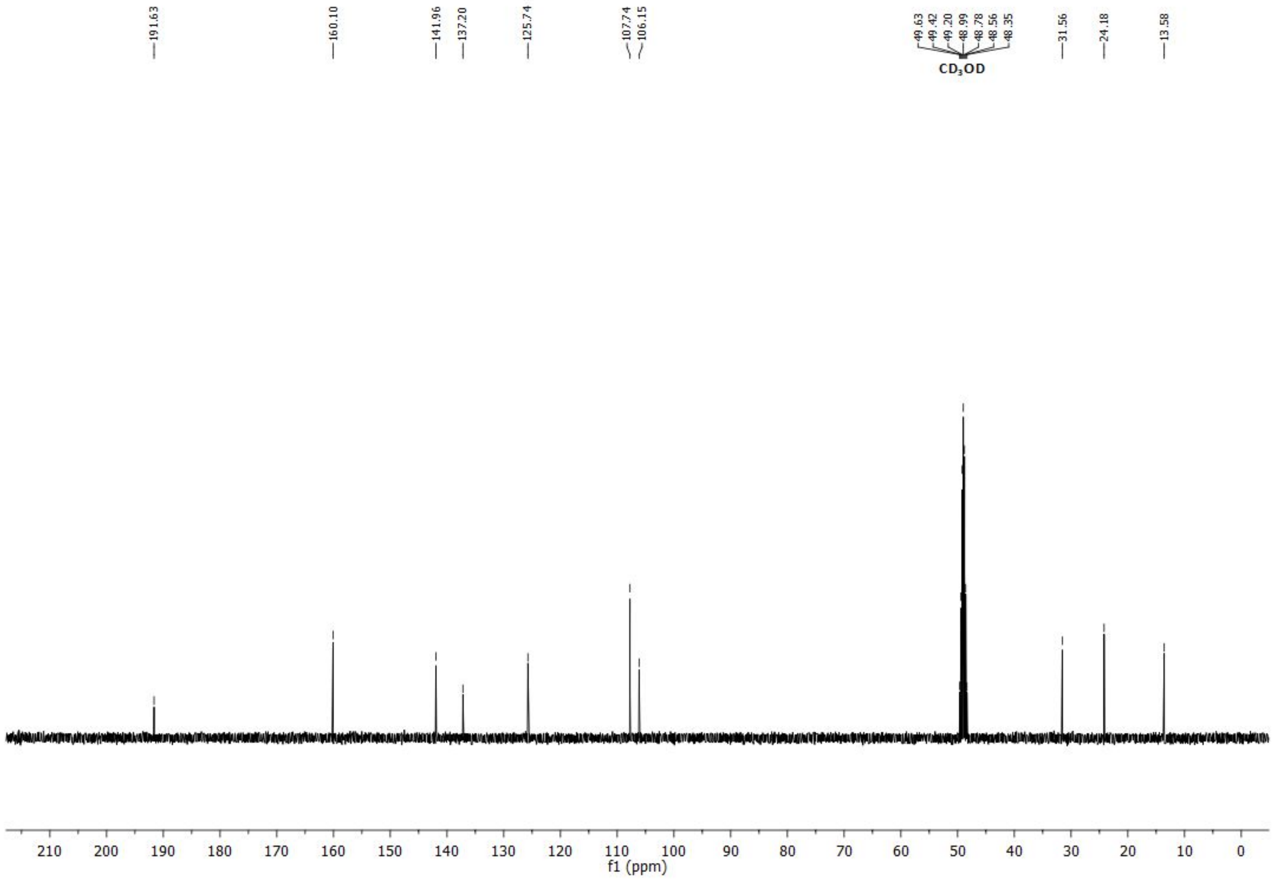

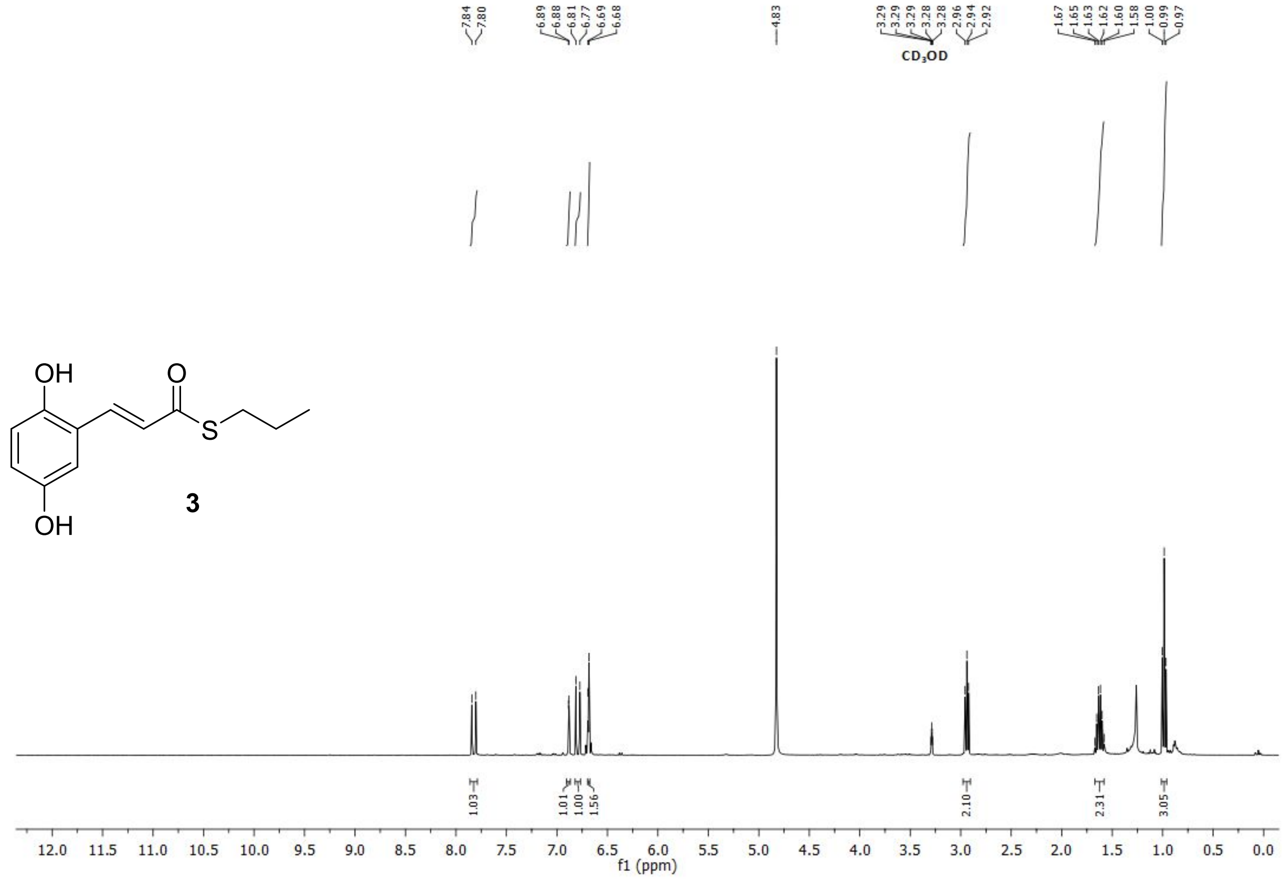
OH
O
S
OH
3
CD₃OD
f1 (ppm)

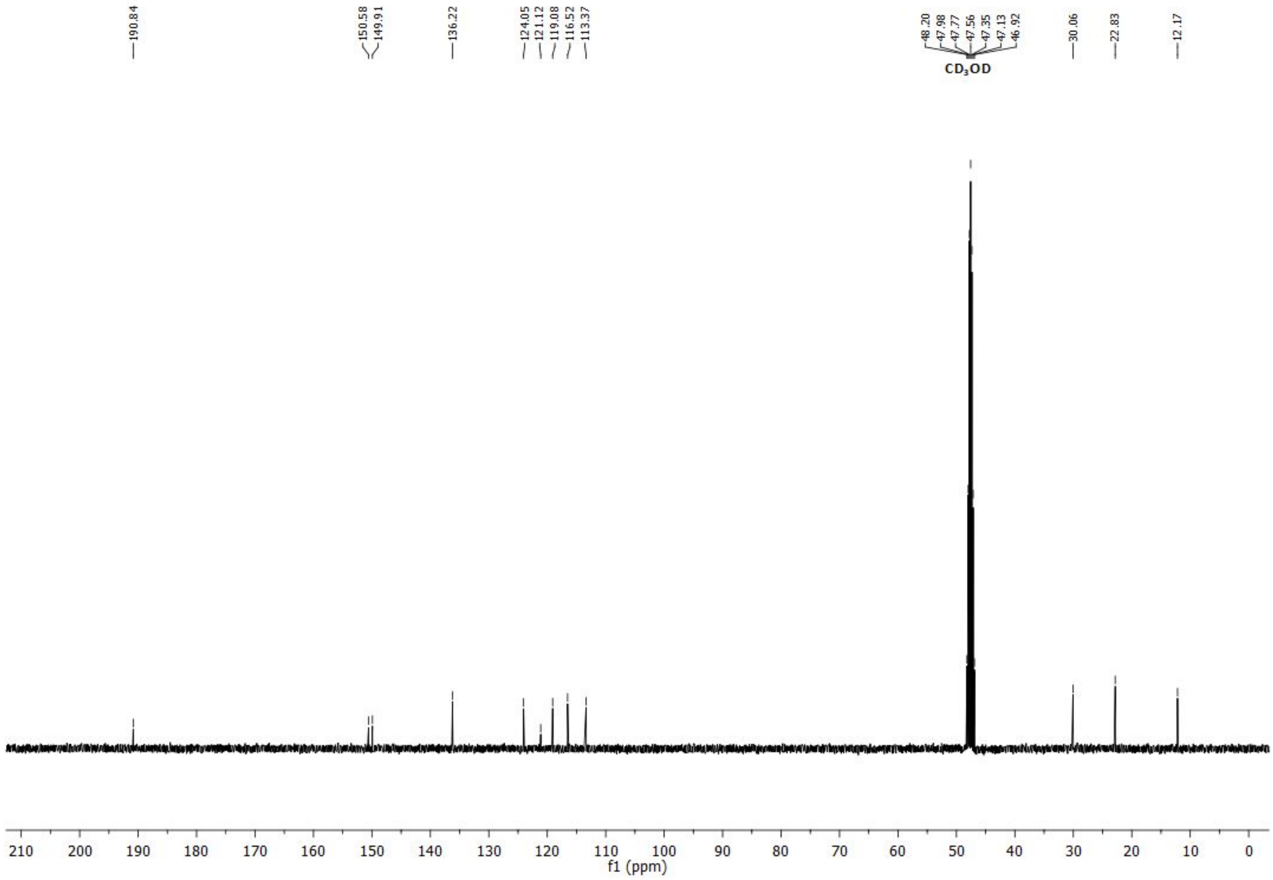
CD₃OD
f1 (ppm)

Optimized geometries of the chemical species shown in **Figure 4**

A) Reactants (R)

*p*-Quinone

| | | | | | |
|---|---|---|---|---|---|
| 1 | 6 | 0 | -4.684107 | 1.003908 | -0.239094 |
| 2 | 6 | 0 | -5.103002 | -0.260884 | -0.149892 |
| 3 | 6 | 0 | -4.140645 | -1.370763 | 0.085160 |
| 4 | 6 | 0 | -2.704422 | -1.022346 | 0.209142 |
| 5 | 6 | 0 | -2.257461 | 0.241559 | 0.093938 |
| 6 | 6 | 0 | -3.246316 | 1.357392 | -0.106912 |
| 7 | 8 | 0 | -2.882593 | 2.514640 | -0.152749 |
| 8 | 8 | 0 | -4.517341 | -2.523095 | 0.172857 |
| 9 | 6 | 0 | -0.850214 | 0.639025 | 0.185380 |
| 10 | 6 | 0 | 0.184903 | -0.177605 | -0.040313 |
| 11 | 6 | 0 | 1.571572 | 0.340353 | 0.101469 |
| 12 | 8 | 0 | 1.842329 | 1.474017 | 0.421638 |
| 13 | 16 | 0 | 2.817860 | -0.884901 | -0.248474 |
| 14 | 6 | 0 | 4.290039 | 0.144688 | 0.046042 |
| 15 | 6 | 0 | 5.558713 | -0.671520 | -0.175006 |
| 16 | 6 | 0 | 6.804255 | 0.178253 | 0.067763 |
| 17 | 1 | 0 | -5.355317 | 1.840665 | -0.404098 |
| 18 | 1 | 0 | -6.146214 | -0.547164 | -0.238046 |
| 19 | 1 | 0 | -2.039454 | -1.857982 | 0.408623 |
| 20 | 1 | 0 | -0.650021 | 1.680716 | 0.423983 |
| 21 | 1 | 0 | 0.060067 | -1.214382 | -0.343142 |
| 22 | 1 | 0 | 4.230698 | 0.527649 | 1.068053 |
| 23 | 1 | 0 | 4.239681 | 1.001619 | -0.630568 |
| 24 | 1 | 0 | 5.568138 | -1.064873 | -1.197307 |
| 25 | 1 | 0 | 5.559549 | -1.536602 | 0.496965 |
| 26 | 1 | 0 | 7.714138 | -0.405425 | -0.089487 |
| 27 | 1 | 0 | 6.822394 | 0.562021 | 1.091924 |
| 28 | 1 | 0 | 6.831532 | 1.035318 | -0.611348 |

Energy: -1088.004018 au
Free energy: -1087.834121 au

Methylamine

| | | | | | |
|---|---|---|---|---|---|
| 1 | 7 | 0 | -0.749150 | -0.000000 | -0.124550 |
| 2 | 6 | 0 | 0.702992 | -0.000000 | 0.017776 |
| 3 | 1 | 0 | 1.079768 | -0.000010 | 1.051895 |
| 4 | 1 | 0 | 1.113671 | 0.878796 | -0.485958 |
| 5 | 1 | 0 | 1.113673 | -0.878784 | -0.485978 |
| 6 | 1 | 0 | -1.140508 | 0.811408 | 0.342615 |
| 7 | 1 | 0 | -1.140505 | -0.811409 | 0.342616 |

Energy: -95.804301 au
Free energy: -95.762357 au

Water

| | | | | | |
|---|---|---|---|---|---|
| 1 | 8 | 0 | 0.000000 | 0.117813 | 0.000000 |
| 2 | 1 | 0 | 0.760185 | -0.471279 | 0.000000 |
| 3 | 1 | 0 | -0.760185 | -0.471228 | 0.000000 |

Energy: -76.383939 au
Free energy: -76.380529 au

B) Attack without water

Pre-reactant state (PR)

| | | | | | |
|---|---|---|---|---|---|
| 1 | 6 | 0 | 4.168281 | -1.588123 | 0.213187 |
| 2 | 6 | 0 | 4.655063 | -0.488526 | -0.365992 |
| 3 | 6 | 0 | 3.750706 | 0.523792 | -0.972876 |
| 4 | 6 | 0 | 2.290774 | 0.272269 | -0.891817 |
| 5 | 6 | 0 | 1.777987 | -0.805225 | -0.273069 |
| 6 | 6 | 0 | 2.707239 | -1.832025 | 0.309820 |
| 7 | 8 | 0 | 2.275159 | -2.836880 | 0.839731 |
| 8 | 8 | 0 | 4.187917 | 1.499508 | -1.553135 |
| 9 | 6 | 0 | 0.345417 | -1.080599 | -0.134818 |
| 10 | 6 | 0 | -0.619103 | -0.157088 | -0.221271 |
| 11 | 7 | 0 | 3.566281 | 1.828020 | 1.467172 |
| 12 | 6 | 0 | 2.619600 | 2.849041 | 1.023266 |
| 13 | 6 | 0 | -2.040092 | -0.563171 | -0.075580 |
| 14 | 8 | 0 | -2.408958 | -1.698369 | 0.115097 |
| 15 | 16 | 0 | -3.179262 | 0.805150 | -0.208327 |
| 16 | 6 | 0 | -4.729862 | -0.120959 | 0.019471 |
| 17 | 6 | 0 | -5.925539 | 0.822226 | -0.052440 |
| 18 | 6 | 0 | -7.235219 | 0.060266 | 0.138968 |
| 19 | 1 | 0 | 4.797604 | -2.354990 | 0.653248 |
| 20 | 1 | 0 | 5.717935 | -0.281483 | -0.443886 |
| 21 | 1 | 0 | 1.664164 | 1.016114 | -1.377188 |
| 22 | 1 | 0 | 0.064255 | -2.107731 | 0.084902 |
| 23 | 1 | 0 | -0.408832 | 0.897699 | -0.381916 |
| 24 | 1 | 0 | 2.638680 | 3.784030 | 1.599384 |
| 25 | 1 | 0 | 1.605782 | 2.440548 | 1.076502 |
| 26 | 1 | 0 | 2.829686 | 3.086918 | -0.023739 |
| 27 | 1 | 0 | -4.676560 | -0.630137 | 0.985178 |
| 28 | 1 | 0 | -4.777212 | -0.889995 | -0.755811 |
| 29 | 1 | 0 | -5.929661 | 1.335335 | -1.020246 |
| 30 | 1 | 0 | -5.828190 | 1.596908 | 0.715943 |
| 31 | 1 | 0 | -8.093121 | 0.734621 | 0.087310 |
| 32 | 1 | 0 | -7.257715 | -0.440508 | 1.111217 |
| 33 | 1 | 0 | -7.359588 | -0.703849 | -0.633829 |
| 34 | 1 | 0 | 3.382018 | 1.571005 | 2.431999 |
| 35 | 1 | 0 | 4.510513 | 2.201631 | 1.439316 |

Energy: -1183.819991 au

Free energy: -1183.591096 au

**Transition state (TS0)**

| | | | | | |
|---|---|---|---|---|---|
| 1 | 6 | 0 | 4.360657 | -1.485164 | 0.096259 |
| 2 | 6 | 0 | 4.813461 | -0.276863 | -0.256841 |
| 3 | 6 | 0 | 3.866930 | 0.802076 | -0.598898 |
| 4 | 6 | 0 | 2.389552 | 0.512731 | -0.439663 |
| 5 | 6 | 0 | 1.945630 | -0.797483 | -0.179500 |
| 6 | 6 | 0 | 2.899265 | -1.836316 | 0.159590 |
| 7 | 8 | 0 | 2.599709 | -2.982949 | 0.468000 |
| 8 | 8 | 0 | 4.236003 | 1.908677 | -0.945237 |
| 9 | 6 | 0 | 0.544889 | -1.081417 | -0.093520 |
| 10 | 6 | 0 | -0.467707 | -0.185941 | -0.214284 |
| 11 | 7 | 0 | 2.045141 | 1.562620 | 1.076453 |
| 12 | 6 | 0 | 1.953148 | 3.011462 | 0.851081 |
| 13 | 6 | 0 | -1.857686 | -0.624289 | -0.072315 |
| 14 | 8 | 0 | -2.224563 | -1.752660 | 0.170209 |
| 15 | 16 | 0 | -3.044289 | 0.721374 | -0.274981 |

| | | | | | |
|---|---|---|---|---|---|
| 16 | 6 | 0 | -4.570000 | -0.235440 | -0.014189 |
| 17 | 6 | 0 | -5.792377 | 0.667695 | -0.132786 |
| 18 | 6 | 0 | -7.082179 | -0.121753 | 0.081221 |
| 19 | 1 | 0 | 5.031082 | -2.300406 | 0.353288 |
| 20 | 1 | 0 | 5.868171 | -0.028823 | -0.322396 |
| 21 | 1 | 0 | 1.784533 | 1.148592 | -1.088977 |
| 22 | 1 | 0 | 0.276698 | -2.112743 | 0.130591 |
| 23 | 1 | 0 | -0.305438 | 0.864974 | -0.449001 |
| 24 | 1 | 0 | 1.731348 | 3.551246 | 1.774859 |
| 25 | 1 | 0 | 1.155780 | 3.195814 | 0.128846 |
| 26 | 1 | 0 | 2.896269 | 3.355061 | 0.425743 |
| 27 | 1 | 0 | -4.511049 | -0.702662 | 0.972140 |
| 28 | 1 | 0 | -4.592490 | -1.038837 | -0.754855 |
| 29 | 1 | 0 | -5.803485 | 1.140581 | -1.120941 |
| 30 | 1 | 0 | -5.723108 | 1.476562 | 0.603004 |
| 31 | 1 | 0 | -7.959218 | 0.524444 | -0.003320 |
| 32 | 1 | 0 | -7.097619 | -0.583749 | 1.072753 |
| 33 | 1 | 0 | -7.178711 | -0.920265 | -0.660210 |
| 34 | 1 | 0 | 1.157689 | 1.154018 | 1.374529 |
| 35 | 1 | 0 | 2.751493 | 1.315539 | 1.767759 |

Energy: -1183,802958 au
Free energy. -1183.568774 au

Intermediate (I1)

| | | | | | |
|---|---|---|---|---|---|
| 1 | 6 | 0 | 4.387502 | -1.469826 | -0.311853 |
| 2 | 6 | 0 | 4.840806 | -0.215426 | -0.449519 |
| 3 | 6 | 0 | 3.934743 | 0.901504 | -0.175263 |
| 4 | 6 | 0 | 2.431272 | 0.633005 | -0.297618 |
| 5 | 6 | 0 | 1.998118 | -0.764850 | -0.030594 |
| 6 | 6 | 0 | 2.960009 | -1.829195 | 0.008502 |
| 7 | 8 | 0 | 2.713944 | -3.011470 | 0.235212 |
| 8 | 8 | 0 | 4.316641 | 1.997908 | 0.197257 |
| 9 | 6 | 0 | 0.627485 | -1.046286 | 0.071504 |
| 10 | 6 | 0 | -0.421366 | -0.180441 | -0.150354 |
| 11 | 7 | 0 | 1.766697 | 1.578687 | 0.714091 |
| 12 | 6 | 0 | 1.450301 | 2.940276 | 0.207300 |
| 13 | 6 | 0 | -1.789312 | -0.612244 | 0.069753 |
| 14 | 8 | 0 | -2.144452 | -1.673519 | 0.537400 |
| 15 | 16 | 0 | -3.010676 | 0.644997 | -0.407964 |
| 16 | 6 | 0 | -4.515732 | -0.273245 | 0.040835 |
| 17 | 6 | 0 | -5.757327 | 0.564956 | -0.241003 |
| 18 | 6 | 0 | -7.030291 | -0.192652 | 0.130832 |
| 19 | 1 | 0 | 5.053519 | -2.324872 | -0.391909 |
| 20 | 1 | 0 | 5.885422 | 0.030234 | -0.610008 |
| 21 | 1 | 0 | 2.098151 | 1.016800 | -1.278017 |
| 22 | 1 | 0 | 0.373429 | -2.057142 | 0.388814 |
| 23 | 1 | 0 | -0.290747 | 0.787254 | -0.633959 |
| 24 | 1 | 0 | 0.983920 | 3.516784 | 1.005759 |
| 25 | 1 | 0 | 0.757068 | 2.841144 | -0.628088 |
| 26 | 1 | 0 | 2.381794 | 3.402078 | -0.111828 |
| 27 | 1 | 0 | -4.444967 | -0.541490 | 1.098012 |
| 28 | 1 | 0 | -4.524497 | -1.204571 | -0.530973 |
| 29 | 1 | 0 | -5.781666 | 0.837638 | -1.301846 |
| 30 | 1 | 0 | -5.702330 | 1.502371 | 0.323701 |
| 31 | 1 | 0 | -7.920980 | 0.407160 | -0.071814 |
| 32 | 1 | 0 | -7.033464 | -0.453380 | 1.193330 |
| 33 | 1 | 0 | -7.112301 | -1.122070 | -0.440302 |

| | | | | | |
|---|---|---|---|---|---|
| 34 | 1 | 0 | 0.897842 | 1.099656 | 1.008024 |
| 35 | 1 | 0 | 2.397428 | 1.654827 | 1.515802 |

Energy: -1183.810064 au
Free energy: -1183.572434 au

Intermediate (I2)

| | | | | | |
|---|---|---|---|---|---|
| 1 | 6 | 0 | 4.241573 | -1.598558 | -0.222647 |
| 2 | 6 | 0 | 4.853606 | -0.403920 | -0.248549 |
| 3 | 6 | 0 | 4.057705 | 0.811514 | 0.000386 |
| 4 | 6 | 0 | 2.802784 | -1.742285 | -0.032189 |
| 5 | 8 | 0 | 2.304484 | -3.003798 | 0.057260 |
| 6 | 6 | 0 | 1.963722 | -0.673286 | 0.019321 |
| 7 | 6 | 0 | 2.539365 | 0.716108 | -0.214527 |
| 8 | 8 | 0 | 4.575304 | 1.846465 | 0.368956 |
| 9 | 6 | 0 | 0.535399 | -0.920597 | 0.101705 |
| 10 | 6 | 0 | -0.468885 | -0.030165 | 0.014400 |
| 11 | 7 | 0 | 1.833755 | 1.780152 | 0.474793 |
| 12 | 6 | 0 | 1.823735 | 3.043852 | -0.258679 |
| 13 | 6 | 0 | -1.859156 | -0.530997 | 0.065412 |
| 14 | 8 | 0 | -2.182715 | -1.691079 | 0.191790 |
| 15 | 16 | 0 | -3.077154 | 0.775891 | -0.085044 |
| 16 | 6 | 0 | -4.576733 | -0.249626 | 0.016710 |
| 17 | 6 | 0 | -5.823708 | 0.620956 | -0.090377 |
| 18 | 6 | 0 | -7.093153 | -0.224036 | -0.007157 |
| 19 | 1 | 0 | 2.420984 | 0.896780 | -1.299723 |
| 20 | 1 | 0 | 4.826860 | -2.511499 | -0.325694 |
| 21 | 1 | 0 | 5.930208 | -0.298335 | -0.321532 |
| 22 | 1 | 0 | 0.248323 | -1.963978 | 0.211317 |
| 23 | 1 | 0 | 1.345251 | 3.805668 | 0.360402 |
| 24 | 1 | 0 | 1.215654 | 2.924509 | -1.162247 |
| 25 | 1 | 0 | 2.820805 | 3.399377 | -0.545719 |
| 26 | 1 | 0 | -4.548325 | -0.794245 | 0.964073 |
| 27 | 1 | 0 | -4.534120 | -0.989518 | -0.786852 |
| 28 | 1 | 0 | -5.804240 | 1.173609 | -1.036098 |
| 29 | 1 | 0 | -5.818833 | 1.367596 | 0.711272 |
| 30 | 1 | 0 | -7.140289 | -0.766037 | 0.941934 |
| 31 | 1 | 0 | -7.125291 | -0.961419 | -0.814631 |
| 32 | 1 | 0 | -7.987370 | 0.399072 | -0.084094 |
| 33 | 1 | 0 | 2.275514 | 1.924619 | 1.378462 |
| 34 | 1 | 0 | 3.021277 | -3.644265 | 0.108900 |
| 35 | 1 | 0 | -0.302336 | 1.036274 | -0.069517 |

Energy: -1183.828158 au
Free energy: -1183.593517 au

## C) Attack assisted by a water molecule

Pre-reactant state (PRw)

| | | | | | |
|---|---|---|---|---|---|
| 1 | 6 | 0 | 4.278104 | -0.710645 | -0.491324 |
| 2 | 6 | 0 | 4.750750 | 0.515400 | -0.249267 |
| 3 | 6 | 0 | 3.856501 | 1.703418 | -0.253781 |
| 4 | 6 | 0 | 2.412190 | 1.468154 | -0.486682 |
| 5 | 6 | 0 | 1.919555 | 0.236148 | -0.700354 |
| 6 | 6 | 0 | 2.847714 | -0.940346 | -0.818509 |
| 7 | 8 | 0 | 2.465200 | -2.008958 | -1.262315 |
| 8 | 8 | 0 | 4.290586 | 2.822419 | -0.053394 |
| 9 | 6 | 0 | 0.501670 | -0.090110 | -0.825759 |
| 10 | 6 | 0 | -0.501346 | 0.572281 | -0.238313 |

| | | | | | |
|---|---|---|---|---|---|
| 11 | 7 | 0 | 2.246351 | -1.226845 | 1.701941 |
| 12 | 6 | 0 | 2.133594 | -0.042308 | 2.535726 |
| 13 | 6 | 0 | -1.872533 | 0.019338 | -0.348773 |
| 14 | 8 | 0 | -2.108693 | -1.117044 | -0.713232 |
| 15 | 16 | 0 | -3.153419 | 1.152962 | 0.114961 |
| 16 | 6 | 0 | -4.591403 | 0.064347 | -0.140598 |
| 17 | 6 | 0 | -5.875168 | 0.791517 | 0.244304 |
| 18 | 6 | 0 | -7.093379 | -0.105152 | 0.033592 |
| 19 | 1 | 0 | 4.908723 | -1.594071 | -0.510257 |
| 20 | 1 | 0 | 5.797712 | 0.713070 | -0.041903 |
| 21 | 1 | 0 | 1.777287 | 2.349460 | -0.453860 |
| 22 | 1 | 0 | 0.267970 | -0.989950 | -1.385992 |
| 23 | 1 | 0 | -0.351552 | 1.475634 | 0.347638 |
| 24 | 1 | 0 | 1.885431 | -0.238869 | 3.587591 |
| 25 | 1 | 0 | 1.355549 | 0.609077 | 2.126355 |
| 26 | 1 | 0 | 3.075814 | 0.518193 | 2.509715 |
| 27 | 1 | 0 | -4.439693 | -0.833754 | 0.463425 |
| 28 | 1 | 0 | -4.600779 | -0.239455 | -1.190358 |
| 29 | 1 | 0 | -5.974485 | 1.703359 | -0.354523 |
| 30 | 1 | 0 | -5.818145 | 1.105025 | 1.292257 |
| 31 | 1 | 0 | -8.013947 | 0.413946 | 0.309672 |
| 32 | 1 | 0 | -7.021848 | -1.011650 | 0.641486 |
| 33 | 1 | 0 | -7.178794 | -0.409595 | -1.013522 |
| 34 | 1 | 0 | 1.352428 | -1.701700 | 1.564634 |
| 35 | 1 | 0 | 2.890551 | -1.898114 | 2.107542 |
| 36 | 8 | 0 | 0.118095 | -2.865592 | 0.402624 |
| 37 | 1 | 0 | 0.776296 | -2.882824 | -0.306159 |
| 38 | 1 | 0 | -0.677277 | -2.486116 | 0.002246 |

Energy: -1260.229686 au
Free energy: -1259.974897 au

Transition state (TS0w)

| | | | | | |
|---|---|---|---|---|---|
| 1 | 6 | 0 | 4.273167 | -1.386954 | -0.281871 |
| 2 | 6 | 0 | 4.780983 | -0.156524 | -0.413218 |
| 3 | 6 | 0 | 3.879884 | 1.000265 | -0.620894 |
| 4 | 6 | 0 | 2.402738 | 0.718901 | -0.615121 |
| 5 | 6 | 0 | 1.915787 | -0.578586 | -0.716516 |
| 6 | 6 | 0 | 2.799152 | -1.663887 | -0.319963 |
| 7 | 8 | 0 | 2.406175 | -2.775960 | 0.026614 |
| 8 | 8 | 0 | 4.292601 | 2.137006 | -0.740071 |
| 9 | 6 | 0 | 0.501891 | -0.828334 | -0.671145 |
| 10 | 6 | 0 | -0.505900 | 0.068477 | -0.754218 |
| 11 | 7 | 0 | 2.098976 | 1.277056 | 1.303572 |
| 12 | 6 | 0 | 1.677029 | 2.670548 | 1.424928 |
| 13 | 6 | 0 | -1.840293 | -0.366280 | -0.337568 |
| 14 | 8 | 0 | -2.044784 | -1.343273 | 0.366042 |
| 15 | 16 | 0 | -3.175534 | 0.685782 | -0.881854 |
| 16 | 6 | 0 | -4.552120 | -0.153380 | -0.035279 |
| 17 | 6 | 0 | -5.852322 | 0.611375 | -0.257261 |
| 18 | 6 | 0 | -7.021458 | -0.079847 | 0.441134 |
| 19 | 1 | 0 | 4.897976 | -2.257422 | -0.104023 |
| 20 | 1 | 0 | 5.844462 | 0.057302 | -0.374830 |
| 21 | 1 | 0 | 1.816547 | 1.560933 | -0.976622 |
| 22 | 1 | 0 | 0.224750 | -1.858046 | -0.451385 |
| 23 | 1 | 0 | -0.371763 | 1.104309 | -1.053064 |
| 24 | 1 | 0 | 1.571149 | 2.990942 | 2.466000 |
| 25 | 1 | 0 | 0.714983 | 2.789022 | 0.922671 |

| | | | | | |
|---|---|---|---|---|---|
| 26 | 1 | 0 | 2.415560 | 3.302413 | 0.924644 |
| 27 | 1 | 0 | -4.301195 | -0.220735 | 1.026412 |
| 28 | 1 | 0 | -4.617132 | -1.173065 | -0.422796 |
| 29 | 1 | 0 | -6.053140 | 0.689779 | -1.331360 |
| 30 | 1 | 0 | -5.743564 | 1.634446 | 0.118973 |
| 31 | 1 | 0 | -7.953455 | 0.468053 | 0.283726 |
| 32 | 1 | 0 | -6.848006 | -0.147886 | 1.519095 |
| 33 | 1 | 0 | -7.159087 | -1.095922 | 0.060077 |
| 34 | 1 | 0 | 1.399660 | 0.584029 | 1.606861 |
| 35 | 1 | 0 | 2.953410 | 1.097065 | 1.826451 |
| 36 | 8 | 0 | 0.572066 | -1.189218 | 2.145199 |
| 37 | 1 | 0 | 1.077976 | -1.911472 | 1.744381 |
| 38 | 1 | 0 | -0.313131 | -1.288714 | 1.759117 |

Energy: -1260.211592 au
Free energy: -1259.954709 au

Intermediate (I1w)

| | | | | | |
|---|---|---|---|---|---|
| 1 | 6 | 0 | 4.460097 | -1.304425 | -0.379670 |
| 2 | 6 | 0 | 4.833133 | -0.024015 | -0.521346 |
| 3 | 6 | 0 | 3.845654 | 1.041145 | -0.326301 |
| 4 | 6 | 0 | 2.360515 | 0.678404 | -0.445100 |
| 5 | 6 | 0 | 2.017472 | -0.762882 | -0.290267 |
| 6 | 6 | 0 | 3.042286 | -1.758172 | -0.156304 |
| 7 | 8 | 0 | 2.854336 | -2.951274 | 0.074865 |
| 8 | 8 | 0 | 4.147496 | 2.178710 | -0.008043 |
| 9 | 6 | 0 | 0.663865 | -1.109963 | -0.245158 |
| 10 | 6 | 0 | -0.387381 | -0.305581 | -0.643532 |
| 11 | 7 | 0 | 1.657170 | 1.504508 | 0.642858 |
| 12 | 6 | 0 | 1.020438 | 2.761055 | 0.175332 |
| 13 | 6 | 0 | -1.708379 | -0.564744 | -0.154616 |
| 14 | 8 | 0 | -1.953934 | -1.205869 | 0.871166 |
| 15 | 16 | 0 | -3.040293 | 0.190575 | -1.092034 |
| 16 | 6 | 0 | -4.426004 | -0.208127 | 0.019589 |
| 17 | 6 | 0 | -5.722239 | 0.387420 | -0.518446 |
| 18 | 6 | 0 | -6.900898 | 0.057413 | 0.394984 |
| 19 | 1 | 0 | 5.188367 | -2.110978 | -0.399943 |
| 20 | 1 | 0 | 5.866849 | 0.286969 | -0.632798 |
| 21 | 1 | 0 | 2.010118 | 1.113488 | -1.394898 |
| 22 | 1 | 0 | 0.427317 | -2.083560 | 0.186125 |
| 23 | 1 | 0 | -0.241456 | 0.524950 | -1.328704 |
| 24 | 1 | 0 | 0.588912 | 3.273645 | 1.035080 |
| 25 | 1 | 0 | 0.231573 | 2.506060 | -0.530767 |
| 26 | 1 | 0 | 1.786297 | 3.377964 | -0.292949 |
| 27 | 1 | 0 | -4.188753 | 0.181590 | 1.013032 |
| 28 | 1 | 0 | -4.491314 | -1.295528 | 0.102251 |
| 29 | 1 | 0 | -5.911728 | 0.001037 | -1.525959 |
| 30 | 1 | 0 | -5.614928 | 1.473705 | -0.612995 |
| 31 | 1 | 0 | -7.830377 | 0.483698 | 0.009955 |
| 32 | 1 | 0 | -6.738887 | 0.454809 | 1.401381 |
| 33 | 1 | 0 | -7.036566 | -1.024599 | 0.481597 |
| 34 | 1 | 0 | 0.963789 | 0.898397 | 1.158799 |
| 35 | 1 | 0 | 2.378944 | 1.769588 | 1.318586 |
| 36 | 8 | 0 | 0.089498 | 0.026725 | 2.418182 |
| 37 | 1 | 0 | 0.653957 | -0.713709 | 2.671201 |
| 38 | 1 | 0 | -0.688134 | -0.409803 | 2.009206 |

Energy: -1260.223321 au
Free energy: -1259.962719 au

Intermediate (I2w)

| | | | | | |
|---|---|---|---|---|---|
| 1 | 6 | 0 | 4.249954 | -1.410953 | -0.279122 |
| 2 | 6 | 0 | 4.754089 | -0.177732 | -0.431374 |
| 3 | 6 | 0 | 3.845119 | 0.962317 | -0.672928 |
| 4 | 6 | 0 | 2.363147 | 0.761028 | -0.356105 |
| 5 | 6 | 0 | 1.893013 | -0.673690 | -0.374743 |
| 6 | 6 | 0 | 2.809966 | -1.671980 | -0.295159 |
| 7 | 8 | 0 | 2.538954 | -2.991153 | -0.234356 |
| 8 | 8 | 0 | 4.259660 | 2.047915 | -1.030227 |
| 9 | 6 | 0 | 0.474016 | -0.925558 | -0.264464 |
| 10 | 6 | 0 | -0.508917 | -0.035429 | -0.492451 |
| 11 | 7 | 0 | 2.060532 | 1.314613 | 0.978300 |
| 12 | 6 | 0 | 2.255786 | 2.755969 | 1.113535 |
| 13 | 6 | 0 | -1.889217 | -0.410700 | -0.136510 |
| 14 | 8 | 0 | -2.165346 | -1.356679 | 0.578383 |
| 15 | 16 | 0 | -3.137610 | 0.657570 | -0.818375 |
| 16 | 6 | 0 | -4.603675 | -0.130285 | -0.079706 |
| 17 | 6 | 0 | -5.863861 | 0.636609 | -0.465264 |
| 18 | 6 | 0 | -7.105372 | -0.009634 | 0.145807 |
| 19 | 1 | 0 | 4.886299 | -2.281534 | -0.152195 |
| 20 | 1 | 0 | 5.820252 | 0.019927 | -0.452809 |
| 21 | 1 | 0 | 1.810215 | 1.365305 | -1.085222 |
| 22 | 1 | 0 | 0.141994 | -1.890945 | 0.119521 |
| 23 | 1 | 0 | -0.328935 | 0.961234 | -0.882665 |
| 24 | 1 | 0 | 1.967348 | 3.045186 | 2.126156 |
| 25 | 1 | 0 | 1.590450 | 3.265260 | 0.410105 |
| 26 | 1 | 0 | 3.277846 | 3.100191 | 0.916113 |
| 27 | 1 | 0 | -4.460907 | -0.151189 | 1.003730 |
| 28 | 1 | 0 | -4.642301 | -1.165629 | -0.427501 |
| 29 | 1 | 0 | -5.956555 | 0.665458 | -1.556432 |
| 30 | 1 | 0 | -5.778062 | 1.675052 | -0.127443 |
| 31 | 1 | 0 | -8.009174 | 0.538608 | -0.129719 |
| 32 | 1 | 0 | -7.040397 | -0.026061 | 1.237601 |
| 33 | 1 | 0 | -7.218845 | -1.041746 | -0.198365 |
| 34 | 1 | 0 | 2.605718 | 0.806964 | 1.674155 |
| 35 | 8 | 0 | 0.305782 | -0.554027 | 2.593027 |
| 36 | 1 | 0 | -0.546023 | -0.863741 | 2.260773 |
| 37 | 1 | 0 | 0.515549 | 0.212116 | 2.038478 |
| 38 | 1 | 0 | 1.586872 | -3.146068 | -0.252107 |

Energy: -1260.231856 au

Free energy: -1259.973534 au

Optimized geometries for the chemical species in **Figure S2**.

A) Attack without wáter

Pre-reactant state (PR)

| | | | | | |
|---|---|---|---|---|---|
| 1 | 6 | 0 | -3.943357 | -2.284669 | 0.191102 |
| 2 | 6 | 0 | -4.768881 | -1.252141 | 0.002720 |
| 3 | 6 | 0 | -4.236587 | 0.123734 | -0.195132 |
| 4 | 6 | 0 | -2.766487 | 0.304667 | -0.203560 |
| 5 | 6 | 0 | -1.918975 | -0.726656 | -0.022728 |
| 6 | 6 | 0 | -2.467156 | -2.105433 | 0.210418 |
| 7 | 8 | 0 | -1.731372 | -3.050748 | 0.414235 |
| 8 | 8 | 0 | -4.987454 | 1.070751 | -0.338458 |
| 9 | 6 | 0 | -0.456789 | -0.602790 | -0.009110 |
| 10 | 6 | 0 | 0.194155 | 0.513699 | -0.358725 |
| 11 | 7 | 0 | -1.497401 | 3.422997 | -0.224187 |
| 12 | 6 | 0 | -0.709205 | 3.460536 | 1.010078 |
| 13 | 6 | 0 | 1.664728 | 0.683053 | -0.274784 |
| 14 | 8 | 0 | 2.174892 | 1.782500 | -0.248856 |
| 15 | 16 | 0 | 2.645184 | -0.810726 | -0.229599 |
| 16 | 6 | 0 | 4.278758 | -0.031755 | -0.027925 |
| 17 | 6 | 0 | 5.363915 | -1.101475 | 0.028230 |
| 18 | 6 | 0 | 6.745412 | -0.471093 | 0.191249 |
| 19 | 1 | 0 | -4.290501 | -3.301246 | 0.346395 |
| 20 | 1 | 0 | -5.849652 | -1.352451 | -0.009189 |
| 21 | 1 | 0 | -2.414164 | 1.325543 | -0.355675 |
| 22 | 1 | 0 | 0.081420 | -1.489973 | 0.317096 |
| 23 | 1 | 0 | -0.333780 | 1.413184 | -0.675788 |
| 24 | 1 | 0 | -0.714300 | 4.428365 | 1.528431 |
| 25 | 1 | 0 | 0.326153 | 3.197243 | 0.777719 |
| 26 | 1 | 0 | -1.093556 | 2.703148 | 1.700382 |
| 27 | 1 | 0 | 4.259680 | 0.567874 | 0.885677 |
| 28 | 1 | 0 | 4.431350 | 0.651168 | -0.867347 |
| 29 | 1 | 0 | 5.332990 | -1.702536 | -0.887024 |
| 30 | 1 | 0 | 5.163724 | -1.783956 | 0.861305 |
| 31 | 1 | 0 | 7.524113 | -1.236266 | 0.230971 |
| 32 | 1 | 0 | 6.801657 | 0.115398 | 1.112958 |
| 33 | 1 | 0 | 6.971374 | 0.197174 | -0.644812 |
| 34 | 1 | 0 | -1.112716 | 4.074379 | -0.901370 |
| 35 | 1 | 0 | -2.450623 | 3.725492 | -0.043190 |

Energy: -1183.821196 au
Free energy: -1183.592145 au

Transition state (TS1)

| | | | | | |
|---|---|---|---|---|---|
| 1 | 6 | 0 | 4.084353 | -1.967612 | 0.262942 |
| 2 | 6 | 0 | 4.777931 | -1.027440 | -0.382799 |
| 3 | 6 | 0 | 4.153851 | 0.269411 | -0.795699 |
| 4 | 6 | 0 | 2.753528 | 0.460715 | -0.482784 |
| 5 | 6 | 0 | 1.980141 | -0.494747 | 0.140119 |
| 6 | 6 | 0 | 2.653731 | -1.768517 | 0.604137 |
| 7 | 8 | 0 | 2.048990 | -2.607368 | 1.243384 |
| 8 | 8 | 0 | 4.843388 | 1.111972 | -1.360394 |
| 9 | 6 | 0 | 0.604335 | -0.390726 | 0.432044 |
| 10 | 6 | 0 | -0.108672 | 0.747980 | 0.017030 |
| 11 | 7 | 0 | 0.358079 | 2.214107 | 0.974854 |
| 12 | 6 | 0 | 0.252424 | 3.489886 | 0.254832 |
| 13 | 6 | 0 | -1.612044 | 0.758431 | 0.211865 |

| | | | | | |
|---|---|---|---|---|---|
| 14 | 8 | 0 | -2.212432 | 1.668856 | 0.745006 |
| 15 | 16 | 0 | -2.403488 | -0.672365 | -0.446153 |
| 16 | 6 | 0 | -4.131364 | -0.193105 | -0.109523 |
| 17 | 6 | 0 | -5.075918 | -1.275266 | -0.622451 |
| 18 | 6 | 0 | -6.532034 | -0.904851 | -0.349033 |
| 19 | 1 | 0 | 4.515082 | -2.910346 | 0.585805 |
| 20 | 1 | 0 | 5.827575 | -1.142931 | -0.637084 |
| 21 | 1 | 0 | 2.343147 | 1.413905 | -0.808546 |
| 22 | 1 | 0 | 0.149251 | -1.126483 | 1.082587 |
| 23 | 1 | 0 | 0.177862 | 1.180773 | -0.950867 |
| 24 | 1 | 0 | 0.633079 | 4.322668 | 0.850028 |
| 25 | 1 | 0 | -0.798563 | 3.653112 | 0.017744 |
| 26 | 1 | 0 | 0.830231 | 3.419318 | -0.669438 |
| 27 | 1 | 0 | -4.320570 | 0.767583 | -0.595647 |
| 28 | 1 | 0 | -4.238596 | -0.046701 | 0.968240 |
| 29 | 1 | 0 | -4.836237 | -2.229209 | -0.140447 |
| 30 | 1 | 0 | -4.921609 | -1.416789 | -1.697639 |
| 31 | 1 | 0 | -7.208730 | -1.679693 | -0.716150 |
| 32 | 1 | 0 | -6.796356 | 0.035034 | -0.842376 |
| 33 | 1 | 0 | -6.709923 | -0.782384 | 0.723295 |
| 34 | 1 | 0 | -0.253727 | 2.194549 | 1.788848 |
| 35 | 1 | 0 | 1.316198 | 1.994039 | 1.248070 |

Energy: -1183.790889 au
Free energy: -1183.556182 au

## Intermediate (I1)

| | | | | | |
|---|---|---|---|---|---|
| 1 | 6 | 0 | 4.160965 | -1.842568 | 0.334339 |
| 2 | 6 | 0 | 4.835850 | -0.800375 | -0.160153 |
| 3 | 6 | 0 | 4.159740 | 0.443766 | -0.659729 |
| 4 | 6 | 0 | 2.735103 | 0.498800 | -0.529426 |
| 5 | 6 | 0 | 1.967298 | -0.565384 | -0.015949 |
| 6 | 6 | 0 | 2.686072 | -1.813144 | 0.458137 |
| 7 | 8 | 0 | 2.080442 | -2.753505 | 0.937385 |
| 8 | 8 | 0 | 4.858168 | 1.335443 | -1.144799 |
| 9 | 6 | 0 | 0.604362 | -0.556621 | 0.177633 |
| 10 | 6 | 0 | -0.112210 | 0.708623 | -0.071798 |
| 11 | 7 | 0 | 0.488700 | 1.807488 | 0.827622 |
| 12 | 6 | 0 | 0.279338 | 3.200249 | 0.366971 |
| 13 | 6 | 0 | -1.594252 | 0.659521 | 0.273974 |
| 14 | 8 | 0 | -2.062074 | 1.351395 | 1.155958 |
| 15 | 16 | 0 | -2.525532 | -0.475945 | -0.689918 |
| 16 | 6 | 0 | -4.171994 | -0.132207 | 0.018692 |
| 17 | 6 | 0 | -5.219557 | -0.996557 | -0.675400 |
| 18 | 6 | 0 | -6.609118 | -0.735659 | -0.098062 |
| 19 | 1 | 0 | 4.641831 | -2.752707 | 0.679838 |
| 20 | 1 | 0 | 5.918874 | -0.802420 | -0.250077 |
| 21 | 1 | 0 | 2.268526 | 1.349873 | -1.025344 |
| 22 | 1 | 0 | 0.100791 | -1.397549 | 0.633782 |
| 23 | 1 | 0 | 0.018450 | 1.102843 | -1.093107 |
| 24 | 1 | 0 | 0.734311 | 3.888440 | 1.078718 |
| 25 | 1 | 0 | -0.791960 | 3.382230 | 0.297197 |
| 26 | 1 | 0 | 0.756003 | 3.312761 | -0.607157 |
| 27 | 1 | 0 | -4.382595 | 0.932861 | -0.107234 |
| 28 | 1 | 0 | -4.126424 | -0.340823 | 1.090295 |
| 29 | 1 | 0 | -4.958215 | -2.053614 | -0.557977 |
| 30 | 1 | 0 | -5.215306 | -0.786069 | -1.750292 |
| 31 | 1 | 0 | -7.360130 | -1.353736 | -0.594731 |

| | | | | | |
|---|---|---|---|---|---|
| 32 | 1 | 0 | -6.896557 | 0.311992 | -0.225871 |
| 33 | 1 | 0 | -6.638069 | -0.964171 | 0.971153 |
| 34 | 1 | 0 | 0.072466 | 1.671765 | 1.752381 |
| 35 | 1 | 0 | 1.503755 | 1.583697 | 0.850036 |

Energy: -1183.794995 au
Free energy: -1183.557624 au

Intermediate (I2)

| | | | | | |
|---|---|---|---|---|---|
| 1 | 6 | 0 | 3.958448 | -2.079732 | 0.215329 |
| 2 | 6 | 0 | 4.760258 | -1.077378 | -0.176249 |
| 3 | 6 | 0 | 4.238177 | 0.261447 | -0.426687 |
| 4 | 6 | 0 | 2.928760 | 0.553235 | -0.290637 |
| 5 | 6 | 0 | 1.988115 | -0.480728 | 0.109296 |
| 6 | 6 | 0 | 2.510856 | -1.865965 | 0.409873 |
| 7 | 8 | 0 | 1.785941 | -2.770211 | 0.788331 |
| 8 | 8 | 0 | 5.196704 | 1.148591 | -0.795420 |
| 9 | 6 | 0 | 0.652311 | -0.324031 | 0.198030 |
| 10 | 6 | 0 | -0.113796 | 0.950460 | -0.082528 |
| 11 | 7 | 0 | 0.456381 | 2.102903 | 0.588999 |
| 12 | 6 | 0 | -0.195808 | 3.356516 | 0.222246 |
| 13 | 6 | 0 | -1.584733 | 0.723095 | 0.322391 |
| 14 | 8 | 0 | -2.021721 | 1.100185 | 1.381690 |
| 15 | 16 | 0 | -2.560701 | -0.164797 | -0.870828 |
| 16 | 6 | 0 | -4.108205 | -0.286894 | 0.085044 |
| 17 | 6 | 0 | -5.180071 | -0.995518 | -0.735654 |
| 18 | 6 | 0 | -6.485473 | -1.105393 | 0.049469 |
| 19 | 1 | 0 | 4.328935 | -3.079108 | 0.414124 |
| 20 | 1 | 0 | 5.827937 | -1.217811 | -0.316659 |
| 21 | 1 | 0 | 2.551024 | 1.558995 | -0.441895 |
| 22 | 1 | 0 | 0.083036 | -1.208482 | 0.477111 |
| 23 | 1 | 0 | -0.091203 | 1.144216 | -1.166904 |
| 24 | 1 | 0 | 0.267163 | 4.173266 | 0.779372 |
| 25 | 1 | 0 | -1.276831 | 3.372554 | 0.416335 |
| 26 | 1 | 0 | -0.032451 | 3.543353 | -0.844004 |
| 27 | 1 | 0 | -4.413410 | 0.726887 | 0.356064 |
| 28 | 1 | 0 | -3.890460 | -0.826422 | 1.010290 |
| 29 | 1 | 0 | -4.827058 | -1.993874 | -1.015539 |
| 30 | 1 | 0 | -5.349459 | -0.446460 | -1.668288 |
| 31 | 1 | 0 | -7.254325 | -1.613331 | -0.537173 |
| 32 | 1 | 0 | -6.865133 | -0.115290 | 0.318125 |
| 33 | 1 | 0 | -6.339121 | -1.670402 | 0.974567 |
| 34 | 1 | 0 | 0.366221 | 1.952005 | 1.591289 |
| 35 | 1 | 0 | 4.791248 | 2.013125 | -0.932920 |

Energy: -1183.834147 au
Free energy: -1183.600620 au

## B) Attack assisted by a water molecule

Pre-reactant state (PRw)

| | | | | | |
|---|---|---|---|---|---|
| 1 | 6 | 0 | 3.391720 | -2.907068 | -0.095120 |
| 2 | 6 | 0 | 4.331218 | -1.972164 | 0.058286 |
| 3 | 6 | 0 | 3.964960 | -0.535423 | 0.153150 |
| 4 | 6 | 0 | 2.535313 | -0.163458 | 0.124999 |
| 5 | 6 | 0 | 1.570199 | -1.096678 | -0.012265 |
| 6 | 6 | 0 | 1.952052 | -2.544796 | -0.169636 |
| 7 | 8 | 0 | 1.111420 | -3.400658 | -0.356330 |
| 8 | 8 | 0 | 4.848883 | 0.305141 | 0.243905 |

| | | | | | |
|---|---|---|---|---|---|
| 9 | 6 | 0 | 0.130779 | -0.821220 | -0.047636 |
| 10 | 6 | 0 | -0.408227 | 0.350742 | 0.310602 |
| 11 | 7 | 0 | 1.226540 | 3.088371 | 0.546549 |
| 12 | 6 | 0 | 0.768632 | 3.296271 | -0.825454 |
| 13 | 6 | 0 | -1.859969 | 0.648368 | 0.213056 |
| 14 | 8 | 0 | -2.271123 | 1.785885 | 0.149354 |
| 15 | 16 | 0 | -2.968989 | -0.755378 | 0.215996 |
| 16 | 6 | 0 | -4.527209 | 0.156915 | -0.017931 |
| 17 | 6 | 0 | -5.705800 | -0.810455 | -0.013360 |
| 18 | 6 | 0 | -7.025486 | -0.066085 | -0.205271 |
| 19 | 1 | 0 | 3.613941 | -3.965621 | -0.183455 |
| 20 | 1 | 0 | 5.392067 | -2.195577 | 0.107509 |
| 21 | 1 | 0 | 2.308293 | 0.899188 | 0.224268 |
| 22 | 1 | 0 | -0.489524 | -1.648092 | -0.385543 |
| 23 | 1 | 0 | 0.197186 | 1.198087 | 0.647250 |
| 24 | 1 | 0 | 0.911409 | 4.316139 | -1.209121 |
| 25 | 1 | 0 | -0.294362 | 3.047804 | -0.895286 |
| 26 | 1 | 0 | 1.314163 | 2.613418 | -1.486100 |
| 27 | 1 | 0 | -4.463060 | 0.705375 | -0.961243 |
| 28 | 1 | 0 | -4.608219 | 0.892837 | 0.785931 |
| 29 | 1 | 0 | -5.722044 | -1.362408 | 0.932682 |
| 30 | 1 | 0 | -5.575767 | -1.551705 | -0.809346 |
| 31 | 1 | 0 | -7.870938 | -0.757960 | -0.201344 |
| 32 | 1 | 0 | -7.035638 | 0.473355 | -1.156890 |
| 33 | 1 | 0 | -7.182777 | 0.663454 | 0.594496 |
| 34 | 1 | 0 | 0.723835 | 3.705955 | 1.175957 |
| 35 | 1 | 0 | 2.218781 | 3.311338 | 0.598706 |
| 36 | 8 | 0 | 4.071224 | 3.016822 | -0.150375 |
| 37 | 1 | 0 | 4.451100 | 2.160368 | 0.101021 |
| 38 | 1 | 0 | 4.237268 | 3.094290 | -1.094220 |

Energy: -1260.224535 au
Free energy: -1259.973903 au

Transition state (TS1w)

| | | | | | |
|---|---|---|---|---|---|
| 1 | 6 | 0 | 3.687016 | -2.517113 | 0.218509 |
| 2 | 6 | 0 | 4.486692 | -1.580861 | -0.298412 |
| 3 | 6 | 0 | 3.961115 | -0.227281 | -0.640541 |
| 4 | 6 | 0 | 2.537939 | -0.017983 | -0.573829 |
| 5 | 6 | 0 | 1.681706 | -0.926159 | -0.004291 |
| 6 | 6 | 0 | 2.242404 | -2.251141 | 0.455609 |
| 7 | 8 | 0 | 1.545470 | -3.080255 | 1.005493 |
| 8 | 8 | 0 | 4.755425 | 0.680256 | -0.912898 |
| 9 | 6 | 0 | 0.312427 | -0.686606 | 0.258744 |
| 10 | 6 | 0 | -0.286161 | 0.493625 | -0.166765 |
| 11 | 7 | 0 | 0.404189 | 2.091145 | 0.770899 |
| 12 | 6 | 0 | 0.273253 | 3.306392 | -0.033720 |
| 13 | 6 | 0 | -1.763221 | 0.682648 | 0.095514 |
| 14 | 8 | 0 | -2.254892 | 1.675035 | 0.585386 |
| 15 | 16 | 0 | -2.734569 | -0.703731 | -0.430291 |
| 16 | 6 | 0 | -4.382723 | -0.036971 | -0.026732 |
| 17 | 6 | 0 | -5.459289 | -1.044916 | -0.415675 |
| 18 | 6 | 0 | -6.852534 | -0.512566 | -0.087298 |
| 19 | 1 | 0 | 4.037187 | -3.500342 | 0.516363 |
| 20 | 1 | 0 | 5.550788 | -1.732491 | -0.452490 |
| 21 | 1 | 0 | 2.195808 | 0.965558 | -0.877010 |
| 22 | 1 | 0 | -0.207865 | -1.365859 | 0.923582 |
| 23 | 1 | 0 | 0.041883 | 0.934500 | -1.112080 |

| | | | | | |
|---|---|---|---|---|---|
| 24 | 1 | 0 | 0.695120 | 4.179293 | 0.471813 |
| 25 | 1 | 0 | -0.783192 | 3.473119 | -0.244896 |
| 26 | 1 | 0 | 0.824985 | 3.167129 | -0.966204 |
| 27 | 1 | 0 | -4.507581 | 0.908912 | -0.560412 |
| 28 | 1 | 0 | -4.407663 | 0.181837 | 1.043846 |
| 29 | 1 | 0 | -5.285895 | -1.988586 | 0.112794 |
| 30 | 1 | 0 | -5.385856 | -1.264480 | -1.486334 |
| 31 | 1 | 0 | -7.623853 | -1.234338 | -0.365542 |
| 32 | 1 | 0 | -7.052382 | 0.418823 | -0.625101 |
| 33 | 1 | 0 | -6.951394 | -0.309228 | 0.982965 |
| 34 | 1 | 0 | -0.152338 | 2.140750 | 1.619302 |
| 35 | 1 | 0 | 1.391660 | 1.925191 | 0.982986 |
| 36 | 8 | 0 | 3.238599 | 2.730005 | 0.354350 |
| 37 | 1 | 0 | 3.852553 | 2.173138 | -0.160870 |
| 38 | 1 | 0 | 3.783260 | 3.422009 | 0.740329 |

Energy: -1260.202359 au
Free energy: -1259.946712 au

## Intermediate (I1w)

| | | | | | |
|---|---|---|---|---|---|
| 1 | 6 | 0 | 3.806883 | -2.320508 | 0.336604 |
| 2 | 6 | 0 | 4.540337 | -1.302763 | -0.129877 |
| 3 | 6 | 0 | 3.924764 | -0.034766 | -0.628468 |
| 4 | 6 | 0 | 2.527263 | -0.007535 | -0.759371 |
| 5 | 6 | 0 | 1.697287 | -0.957890 | -0.128287 |
| 6 | 6 | 0 | 2.328901 | -2.231849 | 0.394097 |
| 7 | 8 | 0 | 1.659940 | -3.133581 | 0.863278 |
| 8 | 8 | 0 | 4.673718 | 0.948473 | -0.853034 |
| 9 | 6 | 0 | 0.371096 | -0.774127 | 0.152998 |
| 10 | 6 | 0 | -0.254906 | 0.534422 | -0.170691 |
| 11 | 7 | 0 | 0.415957 | 1.681507 | 0.587125 |
| 12 | 6 | 0 | 0.134313 | 3.029338 | 0.028155 |
| 13 | 6 | 0 | -1.726018 | 0.577616 | 0.232931 |
| 14 | 8 | 0 | -2.110231 | 1.267670 | 1.154150 |
| 15 | 16 | 0 | -2.775254 | -0.467577 | -0.716282 |
| 16 | 6 | 0 | -4.357278 | -0.062673 | 0.098240 |
| 17 | 6 | 0 | -5.491026 | -0.835169 | -0.568037 |
| 18 | 6 | 0 | -6.828720 | -0.518770 | 0.097441 |
| 19 | 1 | 0 | 4.240126 | -3.245114 | 0.704932 |
| 20 | 1 | 0 | 5.626349 | -1.336017 | -0.152743 |
| 21 | 1 | 0 | 2.108083 | 0.888313 | -1.210060 |
| 22 | 1 | 0 | -0.193868 | -1.532406 | 0.679435 |
| 23 | 1 | 0 | -0.155296 | 0.810132 | -1.230893 |
| 24 | 1 | 0 | 0.637822 | 3.772042 | 0.642554 |
| 25 | 1 | 0 | -0.942742 | 3.187661 | 0.017049 |
| 26 | 1 | 0 | 0.555032 | 3.070043 | -0.976059 |
| 27 | 1 | 0 | -4.510325 | 1.017108 | 0.026646 |
| 28 | 1 | 0 | -4.265265 | -0.319086 | 1.156410 |
| 29 | 1 | 0 | -5.287660 | -1.909553 | -0.506775 |
| 30 | 1 | 0 | -5.532958 | -0.580049 | -1.632402 |
| 31 | 1 | 0 | -7.641560 | -1.071641 | -0.378344 |
| 32 | 1 | 0 | -7.059561 | 0.548058 | 0.026066 |
| 33 | 1 | 0 | -6.812364 | -0.788951 | 1.157156 |
| 34 | 1 | 0 | 0.061772 | 1.626929 | 1.546253 |
| 35 | 1 | 0 | 1.453612 | 1.568735 | 0.591161 |
| 36 | 8 | 0 | 3.020428 | 2.695475 | 0.368884 |
| 37 | 1 | 0 | 3.656074 | 2.185688 | -0.200506 |
| 38 | 1 | 0 | 3.566898 | 3.022545 | 1.090133 |

Energy: -1260.215285 au
Free energy: -1259.954422 au

Intermediate (I2w)

| | | | | | |
|---|---|---|---|---|---|
| 1 | 6 | 0 | -3.710191 | -2.420200 | -0.324722 |
| 2 | 6 | 0 | -4.475283 | -1.419515 | 0.144018 |
| 3 | 6 | 0 | -3.894498 | -0.161333 | 0.610248 |
| 4 | 6 | 0 | -2.555645 | 0.009160 | 0.643665 |
| 5 | 6 | 0 | -1.676419 | -0.954934 | 0.020929 |
| 6 | 6 | 0 | -2.241751 | -2.280481 | -0.419650 |
| 7 | 8 | 0 | -1.538172 | -3.174733 | -0.856109 |
| 8 | 8 | 0 | -4.778039 | 0.786457 | 0.977397 |
| 9 | 6 | 0 | -0.395757 | -0.678352 | -0.288703 |
| 10 | 6 | 0 | 0.233246 | 0.671801 | -0.011815 |
| 11 | 7 | 0 | -0.431518 | 1.749705 | -0.750013 |
| 12 | 6 | 0 | 0.071705 | 3.072349 | -0.362009 |
| 13 | 6 | 0 | 1.724755 | 0.592953 | -0.374673 |
| 14 | 8 | 0 | 2.145337 | 0.989719 | -1.434954 |
| 15 | 16 | 0 | 2.748843 | -0.174601 | 0.854228 |
| 16 | 6 | 0 | 4.326184 | -0.158067 | -0.060380 |
| 17 | 6 | 0 | 5.432924 | -0.770767 | 0.790836 |
| 18 | 6 | 0 | 6.764019 | -0.764403 | 0.042035 |
| 19 | 1 | 0 | -4.126392 | -3.365351 | -0.655485 |
| 20 | 1 | 0 | -5.556552 | -1.505863 | 0.201039 |
| 21 | 1 | 0 | -2.139035 | 0.922533 | 1.049033 |
| 22 | 1 | 0 | 0.188857 | -1.444997 | -0.791389 |
| 23 | 1 | 0 | 0.150975 | 0.903237 | 1.060136 |
| 24 | 1 | 0 | -0.398587 | 3.829658 | -0.992752 |
| 25 | 1 | 0 | 1.160181 | 3.171787 | -0.451630 |
| 26 | 1 | 0 | -0.225434 | 3.272454 | 0.671532 |
| 27 | 1 | 0 | 4.549126 | 0.878725 | -0.324426 |
| 28 | 1 | 0 | 4.180993 | -0.714102 | -0.990017 |
| 29 | 1 | 0 | 5.161193 | -1.796609 | 1.061785 |
| 30 | 1 | 0 | 5.527830 | -0.210271 | 1.727205 |
| 31 | 1 | 0 | 7.558039 | -1.202592 | 0.650876 |
| 32 | 1 | 0 | 7.062127 | 0.255480 | -0.217935 |
| 33 | 1 | 0 | 6.693834 | -1.339876 | -0.885517 |
| 34 | 1 | 0 | -0.226476 | 1.596100 | -1.736518 |
| 35 | 8 | 0 | -3.081158 | 2.835118 | 0.046914 |
| 36 | 1 | 0 | -3.445235 | 3.404077 | -0.638042 |
| 37 | 1 | 0 | -4.318489 | 1.646396 | 0.894161 |
| 38 | 1 | 0 | -2.361306 | 2.323133 | -0.374281 |

Energy: -1260.244187 au
Free energy: -1259.984370 au

Optimized geometries for the chemical species in **Figure S3**.

Transition state for the water-mediated addition to the vinyl position of the *o*-quinone species

| | | | | | |
|---|---|---|---|---|---|
| 1 | 6 | 0 | 3.820304 | -1.559883 | 0.551856 |
| 2 | 6 | 0 | 4.420854 | -0.867393 | -0.694824 |
| 3 | 6 | 0 | 3.514069 | -0.009852 | -1.495548 |
| 4 | 6 | 0 | 2.231889 | 0.153647 | -1.142521 |
| 5 | 6 | 0 | 1.633973 | -0.486303 | 0.054895 |
| 6 | 6 | 0 | 2.429789 | -1.287181 | 0.844740 |
| 7 | 6 | 0 | 0.269253 | -0.233149 | 0.371661 |
| 8 | 6 | 0 | -0.515205 | 0.761047 | -0.213460 |
| 9 | 7 | 0 | -0.192516 | 2.500239 | 0.510222 |
| 10 | 6 | 0 | -0.355986 | 3.581203 | -0.462167 |
| 11 | 6 | 0 | -2.018494 | 0.710064 | 0.006115 |
| 12 | 8 | 0 | -2.704099 | 1.672050 | 0.273581 |
| 13 | 16 | 0 | -2.684404 | -0.909366 | -0.237711 |
| 14 | 6 | 0 | -4.447521 | -0.504194 | -0.005536 |
| 15 | 6 | 0 | -5.295415 | -1.758431 | -0.191067 |
| 16 | 6 | 0 | -6.778766 | -1.449193 | -0.000412 |
| 17 | 1 | 0 | 1.593642 | 0.772145 | -1.766661 |
| 18 | 1 | 0 | -0.127908 | -0.736892 | 1.248623 |
| 19 | 1 | 0 | -0.285497 | 1.093121 | -1.227629 |
| 20 | 1 | 0 | -0.083464 | 4.555674 | -0.047430 |
| 21 | 1 | 0 | -1.396627 | 3.601634 | -0.787610 |
| 22 | 1 | 0 | 0.286697 | 3.381178 | -1.324092 |
| 23 | 1 | 0 | -4.716431 | 0.271965 | -0.726898 |
| 24 | 1 | 0 | -4.570165 | -0.082566 | 0.995388 |
| 25 | 1 | 0 | -4.978538 | -2.524429 | 0.524974 |
| 26 | 1 | 0 | -5.125084 | -2.171236 | -1.191306 |
| 27 | 1 | 0 | -7.386294 | -2.347104 | -0.133504 |
| 28 | 1 | 0 | -7.118821 | -0.701761 | -0.723112 |
| 29 | 1 | 0 | -6.971136 | -1.057557 | 1.002654 |
| 30 | 1 | 0 | -0.847533 | 2.602945 | 1.281638 |
| 31 | 1 | 0 | 0.771834 | 2.427287 | 0.862496 |
| 32 | 8 | 0 | 2.576049 | 2.077411 | 1.357236 |
| 33 | 8 | 0 | 4.546165 | -2.268087 | 1.227567 |
| 34 | 1 | 0 | 3.938101 | 0.453409 | -2.381441 |
| 35 | 1 | 0 | 3.238703 | 2.155036 | 0.661508 |
| 36 | 1 | 0 | 2.015399 | -1.775406 | 1.721737 |
| 37 | 8 | 0 | 5.583239 | -1.019983 | -0.994719 |
| 38 | 1 | 0 | 2.597889 | 1.137326 | 1.590784 |

Energy: -1260.183951 au
Free energy: -1259.928630 au

Transition state for the water-mediated addition to the *o*-quinone ring

| | | | | | |
|---|---|---|---|---|---|
| 1 | 6 | 0 | 3.768235 | -1.360770 | -0.021503 |
| 2 | 6 | 0 | 4.216066 | -0.119962 | -0.834623 |
| 3 | 6 | 0 | 3.177952 | 0.661993 | -1.374350 |
| 4 | 6 | 0 | 1.854505 | 0.540503 | -0.864242 |
| 5 | 6 | 0 | 1.396570 | -0.798663 | -0.347803 |
| 6 | 6 | 0 | 2.321206 | -1.700386 | 0.023327 |
| 7 | 6 | 0 | -0.022566 | -1.066011 | -0.137493 |
| 8 | 6 | 0 | -1.031646 | -0.202615 | -0.333374 |
| 9 | 7 | 0 | 1.717277 | 1.468252 | 0.626965 |
| 10 | 6 | 0 | 1.872162 | 2.906818 | 0.374464 |
| 11 | 6 | 0 | -2.422890 | -0.627028 | -0.032901 |
| 12 | 8 | 0 | -2.720454 | -1.717778 | 0.396595 |

| | | | | | |
|---|---|---|---|---|---|
| 13 | 16 | 0 | -3.631365 | 0.641356 | -0.360116 |
| 14 | 6 | 0 | -5.124164 | -0.277246 | 0.133516 |
| 15 | 6 | 0 | -6.365187 | 0.586423 | -0.062920 |
| 16 | 6 | 0 | -7.626575 | -0.169641 | 0.349056 |
| 17 | 1 | 0 | 1.088131 | 1.057355 | -1.445478 |
| 18 | 1 | 0 | -0.284223 | -2.053903 | 0.239059 |
| 19 | 1 | 0 | -0.888472 | 0.803649 | -0.719623 |
| 20 | 1 | 0 | 1.983734 | 3.457559 | 1.309329 |
| 21 | 1 | 0 | 1.003619 | 3.276854 | -0.174007 |
| 22 | 1 | 0 | 2.771238 | 3.032983 | -0.231678 |
| 23 | 1 | 0 | -5.003024 | -0.576917 | 1.177621 |
| 24 | 1 | 0 | -5.167874 | -1.188123 | -0.468832 |
| 25 | 1 | 0 | -6.437414 | 0.890212 | -1.112803 |
| 26 | 1 | 0 | -6.270573 | 1.504661 | 0.526916 |
| 27 | 1 | 0 | -8.516772 | 0.447474 | 0.208275 |
| 28 | 1 | 0 | -7.581509 | -0.461485 | 1.402270 |
| 29 | 1 | 0 | -7.749256 | -1.079325 | -0.245737 |
| 30 | 1 | 0 | 0.854014 | 1.261329 | 1.133035 |
| 31 | 1 | 0 | 2.534202 | 1.134228 | 1.171049 |
| 32 | 8 | 0 | 4.561824 | -2.013536 | 0.623737 |
| 33 | 1 | 0 | 3.440400 | 1.516103 | -1.987347 |
| 34 | 1 | 0 | 2.051195 | -2.663931 | 0.448966 |
| 35 | 8 | 0 | 5.433079 | 0.095919 | -0.880827 |
| 36 | 8 | 0 | 4.315069 | 0.877544 | 1.810747 |
| 37 | 1 | 0 | 4.519739 | 0.044244 | 2.252593 |
| 38 | 1 | 0 | 4.935585 | 0.879891 | 1.060549 |

Energy: -1260.192348 au

Free energy: -1259.934872 au

**Bibliography**

(1) Simoni, E.; Serafini, M. M.; Caporaso, R.; Marchetti, C.; Racchi, M.; Minarini, A.; Bartolini, M.; Lanni, C.; Rosini, M. Targeting the Nrf2/Amyloid-Beta Liaison in Alzheimer's Disease: A Rational Approach. *ACS Chem Neurosci* **2017**, *8* (7), 1618-1627. DOI: 10.1021/acschemneuro.7b00100.
(2) Mobbili, G.; Crucianelli, E.; Barbon, A.; Marcaccio, M.; Pisani, M.; Dalzini, A.; Ussano, E.; Bortolus, M.; Stipa, P.; Astolfi, P. Liponitroxides: EPR study and their efficacy as antioxidants in lipid membranes. *RSC Adv.* **2015**, *5*, 98955-98966.
(3) Bartolini, M.; Naldi, M.; Fiori, J.; Valle, F.; Biscarini, F.; Nicolau, D. V.; Andrisano, V. Kinetic characterization of amyloid-beta 1-42 aggregation with a multimethodological approach. *Anal Biochem* **2011**, *414* (2), 215-225. DOI: 10.1016/j.ab.2011.03.020. Bartolini, M.; Bertucci, C.; Bolognesi, M. L.; Cavalli, A.; Melchiorre, C.; Andrisano, V. Insight into the kinetic of amyloid beta (1-42) peptide self-aggregation: elucidation of inhibitors' mechanism of action. *Chembiochem* **2007**, *8* (17), 2152-2161. DOI: 10.1002/cbic.200700427.
(4) Naiki, H.; Higuchi, K.; Hosokawa, M.; Takeda, T. Fluorometric determination of amyloid fibrils in vitro using the fluorescent dye, thioflavin T1. *Anal Biochem* **1989**, *177* (2), 244-249. DOI: 10.1016/0003-2697(89)90046-8.
(5) Zhao, Y.; Truhlar, D. G. The M06 suite of density functionals for main group thermochemistry, thermochemical kinetics, noncovalent interactions, excited states, and transition elements: two new functionals and systematic testing of four M06-class functionals and 12 other functionals. *Theor. Chem. Acc.* **2008**, *120*, 215-241.
(6) Curutchet, C.; Orozco, M.; Luque, F. J. Solvation in octanol: parametrization of the continuum MST model. *J. Comput. Chem.* **2001**, *22*, 1180-1193. Soteras, I.; Curutchet, C.; Bidon-Chanal, A.; Orozco, M.; Luque, F. J. Extension of the MST model to the IEF formalism: HF and B3LYP parametrizations. *J. Mol. Struct.* **2005**, *727*, 29-40.
(7) *Gaussian 16*; Gaussian Inc.: Wallington CT, 2016.
(8) Kumar, P.; Nagarajan, A.; Uchil, P. D. Analysis of Cell Viability by the MTT Assay. *Cold Spring Harb Protoc* **2018**, *2018* (6). DOI: 10.1101/pdb.prot095505.
